# The Role of Geography in Human Adaptation

**DOI:** 10.1371/journal.pgen.1000500

**Published:** 2009-06-05

**Authors:** Graham Coop, Joseph K. Pickrell, John Novembre, Sridhar Kudaravalli, Jun Li, Devin Absher, Richard M. Myers, Luigi Luca Cavalli-Sforza, Marcus W. Feldman, Jonathan K. Pritchard

**Affiliations:** 1 Department of Human Genetics, University of Chicago, Chicago, Illinois, United States of America; 2 Department of Human Genetics, University of Michigan, Ann Arbor, Michigan, United States of America; 3 HudsonAlpha Institute for Biotechnology, Huntsville, Alabama, United States of America; 4 Department of Genetics, Stanford University, Stanford, California, United States of America; 5 Department of Biological Sciences, Stanford University, Stanford, California, United States of America; 6 Howard Hughes Medical Institute, University of Chicago, Chicago, Illinois, United States of America; University of Aarhus, Denmark

## Abstract

Various observations argue for a role of adaptation in recent human evolution, including results from genome-wide studies and analyses of selection signals at candidate genes. Here, we use genome-wide SNP data from the HapMap and CEPH-Human Genome Diversity Panel samples to study the geographic distributions of putatively selected alleles at a range of geographic scales. We find that the average allele frequency divergence is highly predictive of the most extreme F_ST_ values across the whole genome. On a broad scale, the geographic distribution of putatively selected alleles almost invariably conforms to population clusters identified using randomly chosen genetic markers. Given this structure, there are surprisingly few fixed or nearly fixed differences between human populations. Among the nearly fixed differences that do exist, nearly all are due to fixation events that occurred outside of Africa, and most appear in East Asia. These patterns suggest that selection is often weak enough that neutral processes—especially population history, migration, and drift—exert powerful influences over the fate and geographic distribution of selected alleles.

## Author Summary

Since the beginning of the study of evolution, people have been fascinated by recent human evolution and adaptation. Despite great progress in our understanding of human history, we still know relatively little about the selection pressures and historical factors that have been important over the past 100,000 years. In that time human populations have spread around the world and adapted in a wide variety of ways to the new environments they have encountered. Here, we investigate the genomic signal of these adaptations using a large set of geographically diverse human populations typed at thousands of genetic markers across the genome. We find that patterns at selected loci are predictable from the patterns found at all markers genome-wide. On the basis of this, we argue that selection has been strongly constrained by the historical relationships and gene flow between populations.

## Introduction

One of the central problems in evolutionary biology is to understand the genetic and ecological mechanisms that drive adaptation. With the advent of large-scale SNP and DNA sequence data it is now possible to study selection and adaptation at a genome-wide scale. In recent years there has been considerable progress in identifying potential signals of selection in a wide variety of species [1]–[4].

In this study, we focus on recent adaptation in human populations. In particular, we examine the role of geography and population history in the spread of selectively favored alleles. The methods that we use provide information about adaptive events that have occurred since the divergence of African and non-African populations—i.e., over the last 50–100 KY [5]–[8]. During this time period the environment and ecology of humans have changed profoundly. Humans have spread out of Africa to colonize almost all of the world's land mass, and in the process have experienced a vast range of new climates, diets and ecosystems [6],[9]. Humans have also encountered new pathogens as they moved around the globe and moved into close proximity with domesticated animals, and as human population densities increased.

These changes in human ecology suggest that there has been ample scope for the action of natural selection in recent human evolution. Moreover, most species, including humans, probably face various additional selection pressures on a persistent basis: e.g., due to sexual competition, viability selection and resistance to evolving pathogens. Hence, it seems reasonable that our genomes would show evidence for recent selection, and there is great interest in understanding what types of environmental pressures and biological processes show the strongest signals of adaptation [1],[10],[11].

Some of the strongest evidence for recent adaptation comes from candidate genes where there is both a strong biological hypothesis for selection as well as evidence for selection from unusual haplotype patterns, homozygosity, or extreme values of F_ST_ [1]. Examples include genes involved in malaria resistance such as *G6PD* and the Duffy antigen gene [12]–[14]; genes involved in lighter skin pigmentation in non-Africans (e.g., *SLC24A5*, *SLC45A2* and *KITLG*) [15]–[21]; and a pair of genes involved in dietary adaptations (*lactase* and *salivary amylase*) [22]–[25].

Recent studies have also cast a wider net to identify signals of selection using genome-wide SNP data [16], [17], [26]–[31], or large-scale resequencing data [32],[33]. Most of these studies report many candidate signals of positive selection. However, for most of the signals detected in this way, we do not yet know how the variation affects phenotypes or the nature of the selective pressures; indeed even the target genes are often uncertain. It is difficult to assess what fraction of the candidate signals are genuinely due to selection, rather than being extreme outliers in the neutral distribution [34]; however, simulations generally show that extreme values of various test statistics are more abundant in the real data than would be expected under neutral models [16],[17],[28],[35]. Some studies have also reported enrichment of selection signals in and around genes, as might be expected if selection is concentrated near genes [16],[31],[36], and a recent study has provided robust genome-wide evidence of selection shaping patterns of diversity [37].

While most recent papers on selection in humans have focused on identifying genes and phenotypes involved in selection, our paper aims to learn more generally about the nature and prevalence of positive selection in humans. We also highlight some of the conceptual and methodological challenges in studies of selection. A separate companion paper focuses more closely on individual selection signals of particular interest [21], and a genome browser of our results is available (http://hgdp.uchicago.edu/).

### Data and Populations Studied

We analyzed genome-wide SNP data from two primary sources, namely, the Human Genome Diversity Panel CEPH (HGDP), and the Phase II HapMap. Together, these two data sets provide the best available combination of dense geographic sampling (HGDP) and dense SNP data (Phase II HapMap) and hence provide complementary information for our analysis.

The HGDP data reported by Li et al. [38] consist of 640,000 autosomal SNPs genotyped in 938 unrelated individuals. These individuals include samples from 53 different human populations. They represent much of the span of human genetic diversity [39],[40], albeit with notable sampling gaps in Africa and elsewhere [41],[42]. Using these samples, Rosenberg et al. [40] identified five major genetic clusters corresponding to native populations from sub-Saharan Africa, west Eurasia, east Asia, Oceania and the Americas. There is also an overall relationship between genetic differentiation and geographic distance [43],[44] suggesting that human population history is likely a complex mixture of population splits and gene flow [45].

The HapMap data consist of over 3 million SNPs genotyped in 210 unrelated individuals [26],[36]. These individuals include 60 Yoruba from Ibadan, Nigeria (YRI), 60 individuals of northwest European ancestry from Utah (CEU) and 90 individuals from east Asia (from Beijing and Tokyo) that we analyzed as a single “analysis panel”(here denoted ASN). For those analyses in which uniform SNP ascertainment is most important, we used a subset of the HapMap data consisting of 900,000 SNPs identified by Perlegen Sciences [46]. These SNPs were detected using array-based resequencing in a multiethnic panel, and subsequently genotyped in the HapMap. This screen should have good power to detect high- F_ST_ SNPs since both alleles of a high- F_ST_ SNP are likely to be present in a multiethnic sample (see Methods for further details). Throughout this paper we consider only the autosomes since the smaller effective population size and the smaller sample sizes in the X chromosome data make it inappropriate to merge the X and autosomal data.

### Overview of the Paper

As noted above, we now know of several genes in which recent selection appears to have been very strong, driving new alleles to high frequencies in particular populations or groups of populations [48]–[50]. Some genome-wide studies have estimated that strong selection, with selection coefficients above 1%, is widespread in the genome (e.g., [16],[47]). Similarly, studies of other organisms have identified cases in which selection has created large allele frequency differences between populations, even in the presence of high rates of gene flow [48],[49],[50]. Together, these studies suggest that selection in humans might be a strong force that allows for local adaptation via large allele frequency shifts at individual loci.

If this were the case, then we might expect to find SNPs whose frequency distributions in the HGDP differ dramatically from neutral patterns. For example, some SNPs might show extreme allele frequency differences between closely related populations due to divergent selective pressures [51]. More broadly, we might expect to find alleles whose geographic distributions differ dramatically from the expectations of neutral population structure, if their frequencies are driven by factors such as diet or climate [24],[52]. However, neutral forces including migration and admixture would tend to work against selection, reducing frequency differences between geographically close populations [53],[54]. Hence it is unclear whether selection pressures in humans are strong enough, and sufficiently divergent over short geographic scales, to produce large frequency differences at individual loci.

In this paper, we begin to answer some of these questions by examining the distributions of potentially selected SNPs at a variety of geographic scales. Our approach combines the complementary strengths of the HGDP and HapMap data sets: we use the HGDP to study the geographic distributions of putatively selected alleles at fine scales, and the much denser HapMap data to study differences between continental populations. We aim to learn whether selection in humans is strong enough to generate highly divergent allele frequencies between closely related populations, and geographic distributions that diverge strongly from neutral patterns. At the largest geographic scales, we ask: How effective has selection been at driving allele frequency differentiation between continental groups?

## Results

At its most basic level, natural selection acts to change allele frequencies in populations. Hence, geographically localized selection will lead to allele frequency differences between populations, both at a selected locus and at other closely linked loci. Throughout this paper, we use extreme allele frequency differences between populations as a tool for identifying candidate signals of selection [55].

**Figure 1:**
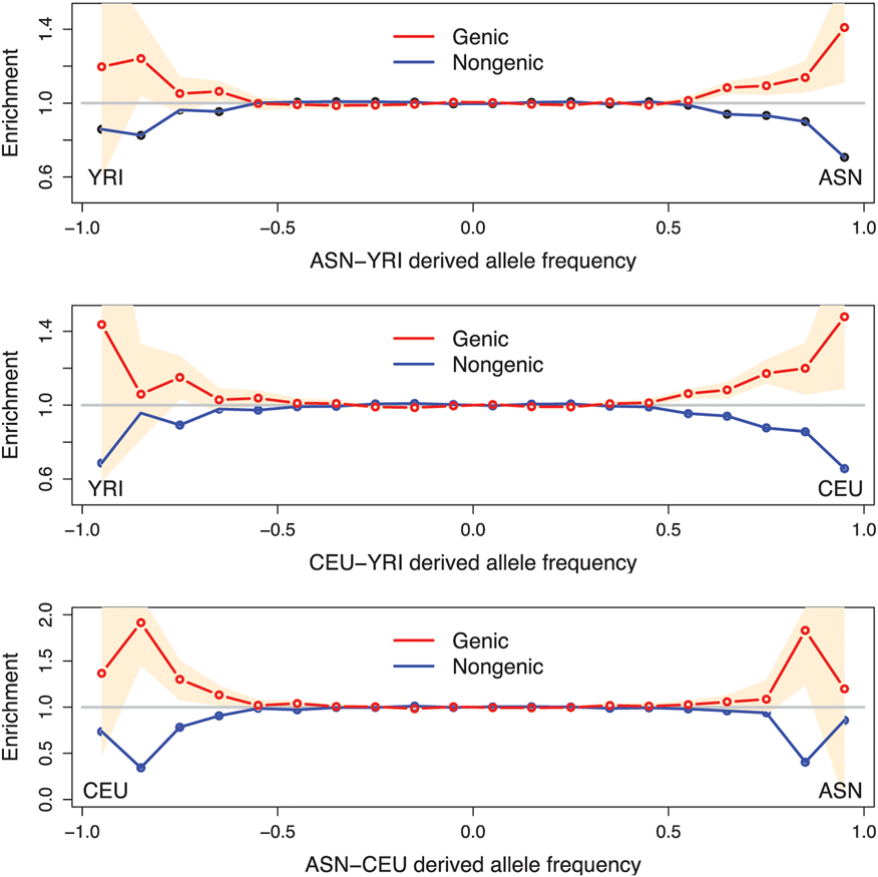
Genic SNPs are more likely than nongenic SNPs to have extreme allele frequency differences between populations. For each plot the x-axis shows the signed difference 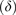 in derived allele frequency between two HapMap populations. The y-axis plots the fold enrichment of genic and nongenic SNPs as a function of 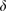: i.e., for each bin we plot the fraction of SNPs in that bin that are genic (respectively, nongenic), divided by the fraction of all SNPs that are genic (respectively, nongenic). The peach-colored region gives the central 90% confidence interval (estimated by bootstrap resampling of 200 kb regions from the genome); when the lower edge of the peach region is >1 this indicates significant enrichment of genic SNPs, assuming a one-tailed test at p = 0.05. Genotype frequencies were estimated from Phase II HapMap data using only SNPs that were identified by Perlegen in a uniform multiethnic panel (“Type A” SNPs) [46]. The numbers of SNPs in the tails are given in Supplementary Table 1 in Text S1.

A major hurdle for any population genetic study of positive selection is to show that the measures used do in fact detect signals of selection rather than just the outliers of a neutral distribution. To test whether the extremes of allele frequency differentiation between populations are enriched for signals of selection, we examined whether large frequency differences between populations are more likely to occur in or near genes (“genic SNPs”) than in non-genic regions. The premise is that genic SNPs are more likely to be functional and so are more likely to be targets of selection. A similar analysis of the HapMap data by Barreiro et al. [31] revealed that the tails of the F_ST_ distribution are enriched for genic variants, and nonsynonymous variants in particular. We extended their analysis to examine the enrichment of genic SNPs in the extremes of frequency differentiation between each pair of HapMap population groups, and included information about the derived allele. To avoid the confounding effects of SNP ascertainment, we used only the subset of SNPs ascertained by resequencing in a multi-ethnic panel (the Perlegen “Type A” SNPs). Figure 1 shows that there is a strong enrichment of genic SNPs in both tails of derived allele frequency differences between all pairs of HapMap populations. There is a similar, perhaps even stronger, enrichment at nonsynonymous sites although, together, nonsynonymous SNPs contribute only a small part of the total genic enrichment (Supplementary Figure 2 in Text S1) [31].

The overall genic enrichment is present in all three population comparisons, and each tail seems to be similarly enriched for high- F_ST_ genic SNPs. However, the number of derived alleles in each tail does differ substantially (see Supplementary Table 1 in Text S1) and is biased towards derived alleles outside Africa and especially in east Asia. Thus, the statistical evidence for enrichment of events inside Africa is weaker than for the other two populations (we return to this point later).

Simulations show that this type of enrichment is expected under models with positive selection and is difficult to explain by other mechanisms (Supplementary Figure 3 in Text S1). One might worry that subtle biases in the Perlegen ascertainment could lead to better detection of high- F_ST_ SNPs in genic regions, but this does not seem to be the case (see Methods). Another reasonable concern is whether models with weakly deleterious mutations could produce this effect either through drift [36] or allelic surfing [56]. However, simulations suggest that models of bottlenecks with weak purifying selection do not inflate F_ST_ in genes (Supplementary Figure 3 in Text S1). Finally, background selection could increase drift in genic regions, thereby increasing the abundance of high- F_ST_ SNPs [57, Supplementary Figure 4 in Text S1]. Theoretical considerations suggest that background selection in humans may be weak [58]; however, direct empirical estimates of the size of this effect are yet to be made, and there is a need for more work on this issue. Thus, in summary, Figure 1 and our simulations strongly suggest that positive selection and associated hitch-hiking are the cause of many of the extreme frequency differences between populations. In light of these results, we will use extremely high- F_ST_ SNPs between these populations as candidate selection signals, while noting that some fraction of these high- F_ST_ SNPs are likely to be drawn from the extreme tail of the neutral distribution.

### Extreme Frequency Differences between Populations as a Function of Mean F_ST_

**Figure 2:**
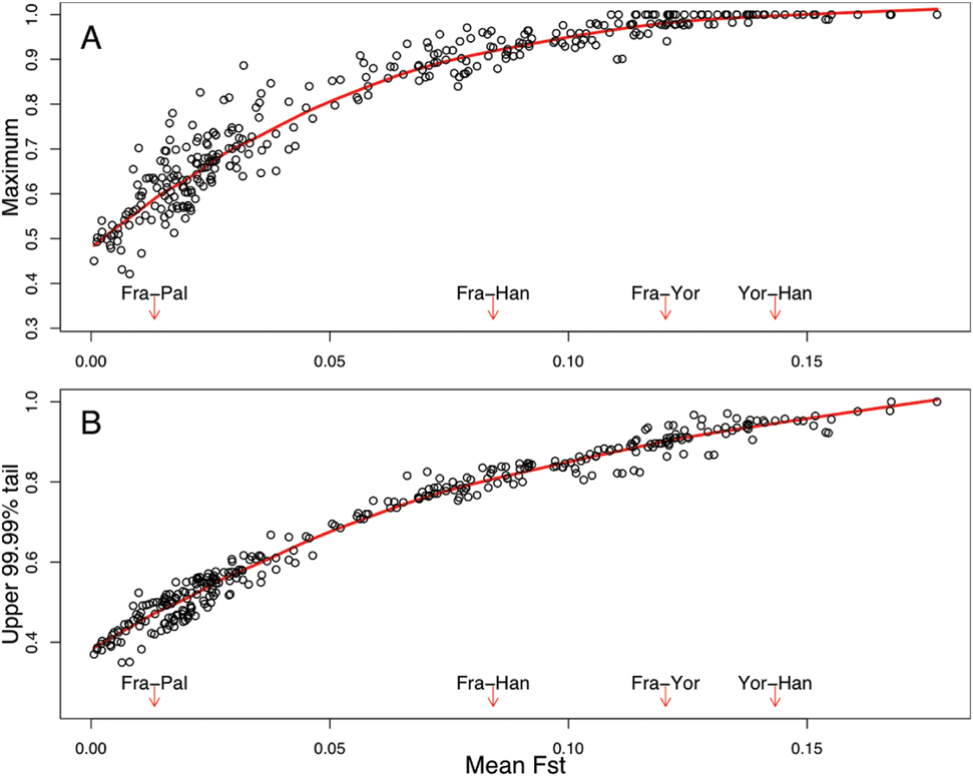
The relationship between mean F_ST_ and the most extreme allele frequency differences genome-wide between pairs of HGDP populations. The x-axis of each plot shows the autosomal mean F_ST_ for pairs of HGDP populations, considering all possible pairs from among the 26 HGDP populations with samples of ≥15 individuals. The y-axes show the value of (A) the maximum autosomal allele frequency difference (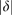) for each population pair, and (B) the value of the 65th most extreme 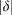 for each population pair (i.e., the 99.99th percentile of the allele frequency distribution). To provide a sense of scale on the figure, red arrows are used to indicate the mean autosomal pairwise F_ST_ between some arbitrary pairs of populations (key: French (Fra), Palestinian (Pal), Han-Chinese (Han) and Yoruba (Yor)). The red lines plot lowess fits to the data. Plots of the extremes of pairwise F_ST_ and with different sample size cutoffs are similar (Supplementary Figures 5 and 6 in Text S1).

Given that a substantial fraction of SNPs with high F_ST_ between the HapMap groups may be targets of selection, we next examined the geographic distributions of high- F_ST_ SNPs across the HGDP. For signals of local adaptation, we searched for examples of SNPs that have highly diverged allele frequencies in pairs of populations that are closely related according to mean F_ST_ (Figure 2). Note that mean F_ST_ between a pair of population is a reasonable proxy for the geographic distance separating the pair [43],[44]. Of course, a possible caveat of studying F_ST_ in the HGDP data is that the Illumina tag SNP panel contains only a subset of all SNPs, and the selected sites might not be included. However, sweeps should usually be detectable because they would change the allele frequencies at nearby tag SNPs; tag SNPs tend to transfer well among the HGDP populations [59]. (Sweeps on standing variation–i.e., existing polymorphisms–are likely to be less-well tagged than sweeps that start from new mutations [60].)

In fact, the data show no examples of SNPs with very extreme allele frequency differences between closely related populations (i.e., population pairs with low mean F_ST_). Moreover, the mean pairwise F_ST_ is highly predictive of the very extreme tail of allele frequency differentiation. If local adaptation were a strong force, we might have expected to find at least some SNPs with extreme frequency differences between closely related populations, or some population pairs with large numbers of high- F_ST_ SNPs. This would be true especially if strong antagonistic selection were widespread: i.e., where different alleles were strongly favored in different locations. Instead, the observation that the extremes of allele frequency differences are so well-predicted by mean F_ST_ might seem consistent with the expectations of an entirely neutral model [61].

However, several observations argue against a fully neutral model for these data. First, simulations show that the tails of differentiation observed here are more extreme than expected under neutral models (see Supplementary Figure 7 in Text S1). Second, as shown in Figure 1, the extremes of allele frequency differences in the HapMap are enriched for genic SNPs, as might be expected if many of these SNPs are selectively favored. This result is also observed at finer geographic scales in the HGDP data (Supplementary Figure 8 in Text S1), although it is unclear whether this result is robust to the Illumina SNP ascertainment scheme. Finally, many of the most extreme SNPs (across a range of mean F_ST_) fall close to strong candidate genes for selection, including skin pigmentation genes, *lactase*, and *Toll-like receptor 6* [21],[22],[62, Supplementary Figure 9 in Text S1]. Although such SNPs with large allele frequency differences are especially strong candidates for being targets of selection, they are not strong outliers from the curves seen in Figure 2, suggesting that they, too, are governed by the predictive relationship between mean F_ST_ and extreme allele frequency differences.

### The Geographic Distributions of High- F_ST_ SNPs

To further investigate the geographic patterns of putatively selected loci, we next focused on the global distributions of SNPs that show extreme differentiation between particular pairs of populations. In the following discussion, we focus on SNPs with extreme pairwise F_ST_ between three HGDP populations: the Yoruba, French and Han Chinese. These three populations were chosen because they are geographically far apart and because there is evidence that selection is responsible for many of the extreme F_ST_ values between each of these groups (Figure 1). Results for additional comparisons are shown in Supplementary Figures 10 and 11 in Text S1.

Under strong selection, the geographic distributions of selected alleles detected in pairwise comparisons might differ greatly from one locus to another. For example, a selected allele that strongly differentiates the French from both the Yoruba and Han could be strongly clinal across Europe, or at high frequency in Europe and absent elsewhere, or follow any other distribution according to the geographic nature of the selective pressure.

**Figure 3:**
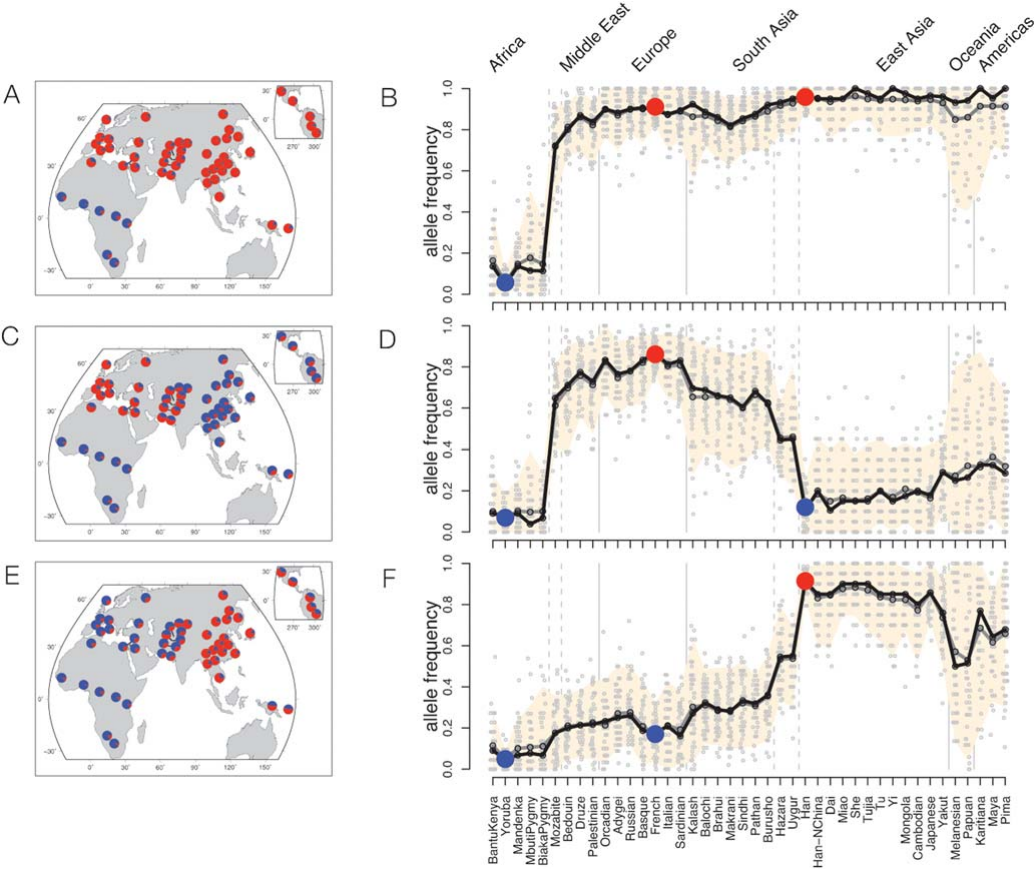
Global allele frequency distributions for SNPs with extreme F_ST_ between certain population pairs. Each row plots frequency distributions for 50 of the most extreme SNPs genome-wide in the following pairs of comparisons: (A, B): SNPs for which Yoruba are highly differentiated from both Han and French; (C, D): French are differentiated from Yoruba and Han; (E, F): Han are differentiated from Yoruba and French. Left column: pie charts of the mean allele frequencies of the 50 highly differentiated SNPs across the HGDP populations; blue and red denote the major and minor alleles in Yoruba, respectively. Right column: The same data are plotted in an expanded format: populations with ≥10 sampled individuals are listed along the x-axis, roughly ordered by geography [40]; vertical grey lines divide the populations based on broad geographic region and dashed grey lines identify populations known to be admixed between broad geographic regions. The y-axis plots allele frequencies in each population; alleles are polarized according to the minor allele in Yoruba. Individual SNP frequencies in each population are shown as grey dots. The mean and median frequencies are shown as gray and black lines, respectively; the peach colored region shows the frequency interval containing the central 94% of the plotted SNP frequencies in each population. SNPs were selected so that each plot includes at most one SNP from clusters of high- F_ST_ SNPs (Methods).

However, we see that the global geographic distributions of these putatively selected alleles are largely determined simply by their frequencies in Yoruba, French and Han (Figure 3). The global distributions fall into three major geographic patterns that we interpret as non-African sweeps, west Eurasian sweeps and East Asian sweeps, respectively. The boundaries of these three patterns are highly concordant with neutral population structure inferred from random microsatellites or SNPs [38],[40]. This is the case even for loci such as *KITLG*, *SLC24A5* and *EDAR* where there is a strong biological case for the genes being targets of selection. Moreover, these patterns are robust to the choice of populations used to identify high- F_ST_ SNPs: for example, very similar results are obtained for SNPs with high F_ST_ between Mandenka, Balochi and Yakut (Supplementary Figure 14 in Text S1).

**Figure 4:**
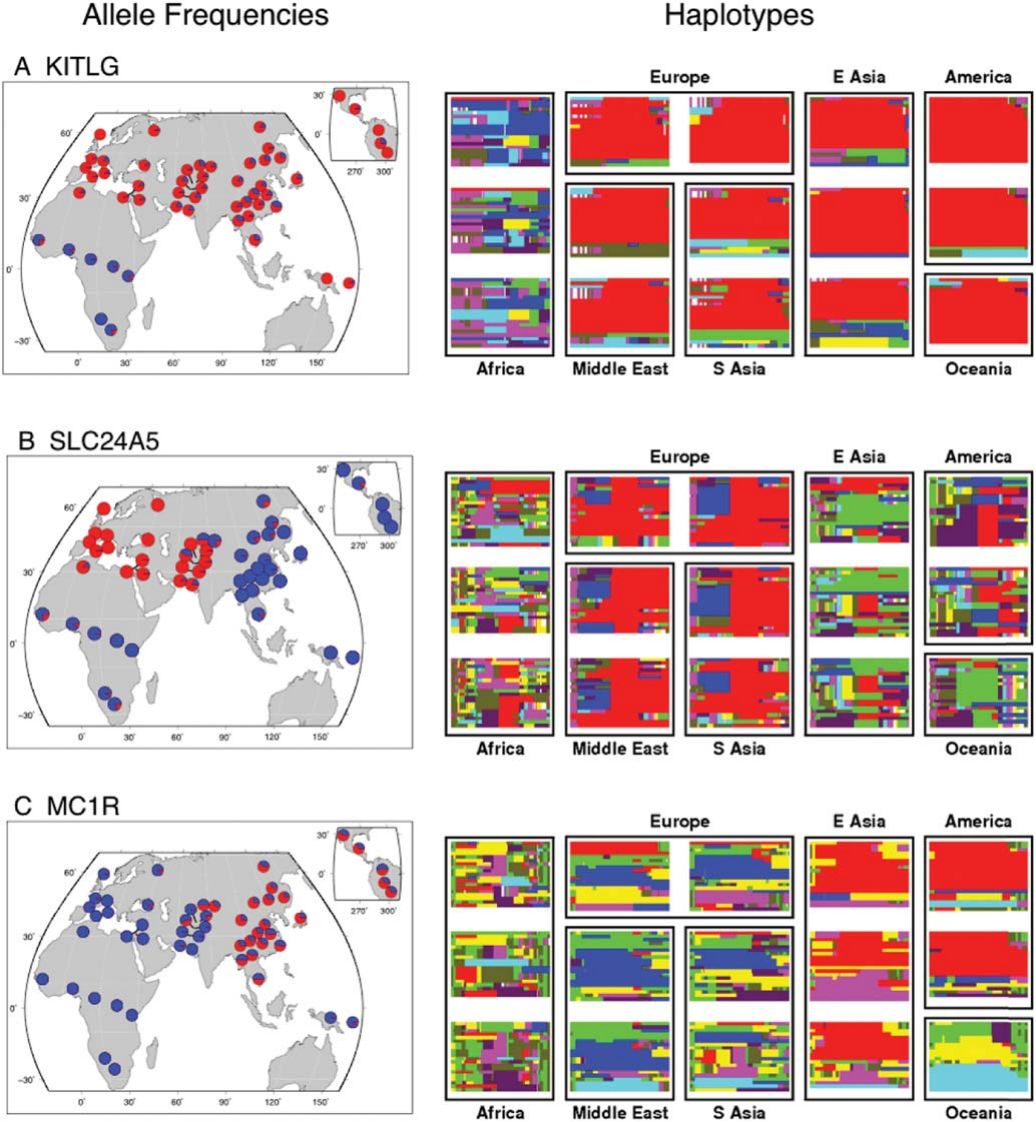
Global allele frequencies and haplotype patterns at three genes with signals of positive selection. The left-hand column shows pie charts of allele frequencies (blue ancestral, red derived) across the HGDP populations for: (A) a SNP upstream of *KITLG* (rs1881227); and for nonsynonymous SNPs in (B) *SLC24A5* (rs1426654; data from [18]), and (C) *MC1R* (rs885479). The right-hand column shows a representation of haplotype patterns for 500 kb around each gene, in each case centered on the SNP displayed in the pie charts. Each box represents a single population, and observed haplotypes are plotted as thin horizontal lines, using the same haplotype coloring for all populations (see Methods and [59]). In all three cases the derived allele plotted in the pie charts is found mainly on the red haplotype.

The first pattern, the “non-African sweep”, is exemplified by a sweep near the *KIT* ligand gene (*KITLG*) (Figure 4A, B). It has been reported previously that HapMap Europeans and East Asians have undergone a selective sweep in the *KITLG* region on a variant that leads to lighter skin pigmentation [20]. Haplotype patterns in the HGDP indicate that a single haplotype has swept almost to fixation in nearly all non-African populations (Figure 4A). More generally, at SNPs that strongly differentiate the HGDP Yoruba from both the Han and French (Figure 3A, B), we observe that typically one allele is rare or absent in all the HGDP Africans, and at uniformly high frequency across Eurasia, the Americas, and usually Oceania. This pattern could be consistent either with sweeps across all the HGDP African populations, or with non-African sweeps that pre-date the colonization of the Americas some 15 KYA [6]. As outlined below, it seems that in fact most of these signals are, like *KITLG*, due to non-African sweeps.

The second pattern, the “west Eurasian sweep” is illustrated by a nonsynonymous SNP in the *SLC24A5* gene (Figure 4C, D). The derived allele at this SNP is also strongly associated with lighter skin color [15],[63] and has clear signals of selection in the HapMap Europeans [15],[17],[35], and in the Middle East and south Asia (Figure 4C). The derived allele is also at high frequency in US-sampled Indian populations [64], supporting the idea that the sampled Indian populations may be similar to the western eurasian HGDP populations at selected as well as neutral SNPs [65]. The derived allele is near fixation in most of the HGDP Eurasian populations west of the Himalayas, and at low frequency elsewhere in the world. More generally, alleles that strongly differentiate the French from both the Han and Yoruba (Figure 3D) are typically present at high frequency across all of Europe, the Middle East and South Asia (an area defined here as “west Eurasia”), and at low frequency elsewhere. This pattern of sharing across the west Eurasian populations is highly consistent with observations from random markers showing that the populations in west Eurasia form a single cluster in some analyses of worldwide population structure [40]. Allele frequencies at high- F_ST_ SNPs in two central Asian populations, the Uygur and Hazara, tend to be intermediate between west Eurasia and east Asia, consistent with observations that these populations have recent mixed ancestry between west Eurasia and east Asia [38],[40],[66].

Finally, the “east Asian sweep” pattern is defined by SNPs that differentiate the Han from French and Yoruba (Figure 3E, F). One example is provided by a nonsynonymous SNP in the *MC1R* gene [67], for which the derived allele is at high frequency in the east Asian and American populations, and virtually absent elsewhere (Figure 4E, F). *MC1R* plays an important role in skin and hair coloration, although the functional impact of this variant in *MC1R*–if any–is unknown [68]. A nonsynonymous SNP in the *EDAR* gene that affects hair morphology shows a very similar geographic pattern [35]. It is interesting that although west Eurasians and east Asians have both evolved towards lighter skin pigmentation, they have done so via largely independent sets of genes [18]. This suggests that favored mutations have not spread freely between the two regions.

It should be noted that rare examples of strong frequency clines within geographic regions do exist, in contrast to the sharp steps seen in Figure 3. For example, SNPs in the *lactase* [22],[69] and *Toll-like receptor 6* [62] gene regions are among the most differentiated SNPs between the French and Palestinian populations (Supplementary Figure 10 in Text S1), and are strongly clinal across Europe. However, these clinal alleles do not appear in Figure 3 because the 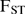 values for these SNPs between the Yoruba, French and Han are less extreme than for the SNPs in Figure 3. We suggest that these alleles may represent relatively recent selection events that have not yet generated extremely large frequency differences between continental groups or had time to disperse more evenly across a broad geographic region.

In summary, we find that the geographic distributions of SNPs with extreme 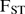 values are highly regular, and agree with population clusters identified using randomly chosen markers. While selected alleles that spread rapidly between geographic locations would not be detectable by 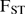 [70], such shared sweeps would be visible from haplotype based signals of selection. Patterns of sharing of haplotype-based signals of selection in the HGDP based on the “integrated haplotype score” (iHS) [16], while somewhat more noisy, support the observation that there is relatively little sharing of partial sweep signals between east Asia, west Eurasia and Africa, but many shared signals within west Eurasia (Supplementary Figure 15 in Text S1; [21]). Thus, the overall distribution of selected alleles is strongly determined by the historical relationships among populations, and suggests again that very local selection pressures (e.g., divergent selection pressures within continental regions) have not given rise to very high- F_ST_ SNPs.

### High- F_ST_ SNPs in the HapMap Populations

**Figure 5:**
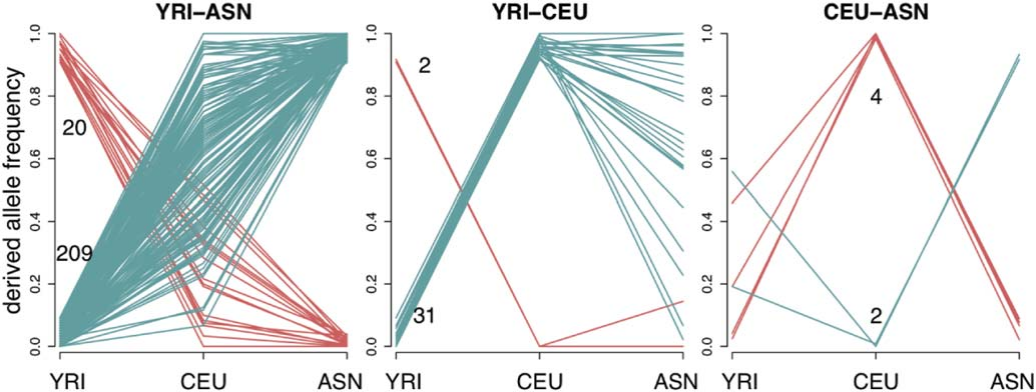
Derived allele frequencies of SNPs with extreme frequency differences between pairs of HapMap populations. In each plot, each red or blue line indicates the derived allele frequencies of a single SNP in the HapMap YRI, CEU, and ASN population groups. The plots show SNPs with extreme frequency differences (>90%) between each pair of HapMap groups: YRI–ASN (left), YRI–CEU (middle), CEU–ASN (right). The data are for Perlegen Type A SNPs genotyped in HapMap. The red lines show alleles that have high derived frequency in the first population and the upper number on each plot indicates the total number of such SNPs; the blue lines and lower numbers are for alleles that are at high frequency in the second population.

Since the allele frequencies of high- F_ST_ SNPs in the Yoruba, French and Han are highly predictive of their frequencies throughout the HGDP, we next turned to the HapMap data–which have much higher SNP density–to further investigate these candidate sweeps. For this analysis, we used Perlegen Type A SNPs that were genotyped in the HapMap [36]. These 900,000 SNPs were identified by screening ∼10% of the genome in a uniform multiethnic panel (see Methods). Figure 5 plots the derived allele frequencies for SNPs with extreme allele frequency differences between each pair of HapMap populations. Results from the full HapMap data are similar (Supplementary Table 3 and Figures 17–20 and in Text S1).

Several interesting points emerge from Figure 5. First, more than 80% of the high- F_ST_ SNPs occur in the Yoruba–east Asia comparison. After clustering together sets of high- F_ST_ SNPs that are tightly linked we again reach a similar result: there are 76 genomic regions with at least one SNP having an allele frequency difference >90% between YRI and ASN, 33 such regions between YRI and CEU, and 6 such regions between CEU and ASN (see Methods for details on the clustering).

Second, the derived allele is almost always at higher frequency in Europeans or east Asians than in Yoruba [36]. This implies that in most cases the sweeps are occurring in the non-African populations. The derived allele is most common in Yoruba at fewer than 10% of the high- F_ST_ SNPs. Even among these few possible examples of sweeps in Yoruba, many seem to be due to hitchhiking of ancestral alleles in non-African sweeps (Supplementary Figure 21 in Text S1). Moreover, simulations show that even if most selection in the Yoruba acted on standing variation, we would still have power to detect about half of all strong YRI sweeps (Supplementary Figure 16 in Text S1). The east Asian bias is unlikely to be due to stronger drift of neutral alleles in the east Asians [71] since the enrichment of genic SNPs is at least as strong in the east Asians as in the other populations (Figure 1).

Third, among the derived alleles that are at low frequency in Yoruba and at high frequency in east Asians, we find that essentially all of these alleles are at intermediate frequency in Europeans (Figure 5A, Supplementary Figure 11 in Text S1). We also observed that for most of these SNPs, the allele frequencies in the Americas are similar to Han frequencies, suggesting that in most cases these alleles were already at high frequency prior to colonization of the Americas some 15,000 years ago (Supplementary Figure 11 in Text S1). Together, the latter observations suggest that perhaps the east Asian sweeps tend to be relatively old. To examine this idea further, we looked at whether the high-frequency high- F_ST_ SNPs in east Asia are surrounded by regions of strongly reduced diversity, as would be expected for recent completed sweeps. Using the XP-EHH measure (cross-population extended haplotype homozygosity) [35], we find that high- F_ST_ SNPs tend to lie in regions of lower variability than random control SNPs. However, the shift in XP-EHH is relatively small, and is far less than for simulated data in which new mutations sweep up with selection coefficients of 1% (see Methods and Supplementary Figures 22 and 23 in Text S1). (But note that strong selection on standing variation would also generate relatively modest XP-EHH signals [60]).

Finally, it is striking just how few SNPs in the genome have extreme allele frequency differences between populations. For example, in the entire Phase II HapMap there are only 13 non-synonymous SNPs with a frequency difference >90% between Yoruba and east Asians (Supplementary Table 5 in Text S1). There are especially few fixation events in the Yoruba: the derived allele is at high frequency in the Yoruba at just one of these 13 nonsynonymous SNPs. These numbers likely represent a substantial fraction of all non-synonymous SNPs in the genome with such extreme frequency differences.

## Discussion

Different analyses of genetic data provide conflicting evidence on the strength and abundance of recent adaptation in humans. An important signal of selection in genome-wide data is that genic (and especially nonsynonymous) SNPs are more likely than nongenic SNPs to have high F_ST_ values between pairs of HapMap populations ([31],[36], Figure 1). This supports the role of positive selection in generating a substantial fraction of the very high- F_ST_ signals. Further support for the action of selection comes from the recent work of [37], and comparisons of genome-wide selection scans with neutral simulations [16],[17],[28],[35]. But in other respects, the data seem to argue that neutral processes–especially population history, migration, and drift–exert powerful influences over the fate and geographic distribution of selected alleles.

We propose below that even if positive selection is common in the genome, strong selection that drives new mutations rapidly to fixation appears to be rare. Our results also argue against a strong form of adaptation in local populations by very large allele frequency shifts at individual loci. However, our data do not preclude a weaker level of adaptive tuning: i.e., modest frequency changes may often occur in response to local conditions [23],[24],[52]. Indeed, it is still possible that small frequency shifts at multiple loci could allow populations to effectively adapt to local conditions even in the absence of large frequency changes at individual loci.

### Geographic Patterns of Selected Variants

Recent studies of humans and other species have shown that populations may adapt to local selection pressures by large frequency changes at relatively few loci [20],[22],[49]. When selection is antagonistic–i.e., different alleles are favored in different environments, as seen for skin pigmentation–then strong selection should generate large allele frequency differences between populations. However, our data show that the geographic distributions of even the highest- F_ST_ SNPs follow patterns that are predictable from neutral variation. Across the entire HGDP data set there are no examples of SNPs with very extreme allele frequency differences between closely related populations, and the distribution of the largest values of allele frequency differentiation between population pairs is accurately predicted by mean F_ST_ (Figure 2). Similarly, at a global scale, the geographic distributions of alleles with high F_ST_ between Yoruba, French and Han, or between Mandenka, Balochi and Yakut, fall into predictable patterns based just on their frequencies in those three populations.

Why is this? First, it is likely that environmental pressures often vary smoothly with geographic distance, and so closely related populations would usually experience similar pressures. Nonetheless, there should be cases in which pairs of closely related populations do face sharply divergent selective pressures due to differences in diet, climate, pathogens or other factors [23],[24],[52]. Similarly, although there should be sets of populations that share particular selective pressures despite not being closely related, the data do not provide obvious examples of this. For example, recall that within Eurasia, the geographic distribution of the skin pigmentation locus *SLC24A5* agrees with population structure estimated from neutral markers, rather than with latitude or climate (Figure 3B).

Our results therefore suggest that local adaptation is tightly constrained by the ancestral relationships and migration rates among populations. It seems likely that selection in humans is generally not divergent enough to generate large frequency differences at individual loci between population pairs that are either recently separated, or regularly exchange migrants [53],[54]. Furthermore, populations may be too mobile, or their identities too fluid, to experience very localized pressures consistently over the several thousand years that may be required for large allele frequency changes.

However in contrast, it seems that selected alleles may not spread effectively *between* broad geographic regions (see Figure 3, Supplementary Figure 15 in Text S1 and [21]). Perhaps this is because populations usually adapt to similar selection pressures by parallel mutation [18],[23],[25] rather than by the spread of migrants between regions [72],[73].

In summary, we propose that the strongest determinants of the geographic distribution of favored variants may be the times at which they first spread to intermediate frequencies and the subsequent history of population movements and range expansions, population splitting and exchange of migrants. We suggest that variants that are broadly distributed across the non-African populations (such as the *KITLG* mutation) typically reached intermediate frequencies shortly after the out-of-Africa migration, and subsequently spread around the globe as populations expanded. At the other extreme, we suggest that local, strongly clinal patterns (as seen in Europe at *lactase* and *Toll-like receptor 6* [62]) may usually indicate that these alleles have spread to intermediate frequency comparatively recently. These hypotheses will need to be tested by future studies.

### SNPs with High- F_ST_ between Continental Groups

**Figure 6:**
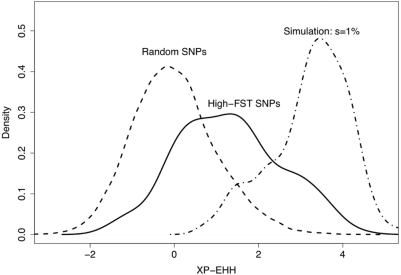
The distribution of XP-EHH, a measure of haplotype homozygosity, at high- F_ST_ SNPs in east Asians. The solid line shows the distribution of XP-EHH [35] in the ASN population at SNPs with a frequency difference >90% between the ASN and YRI samples. For comparison, we plot the XP-EHH distribution both for SNPs randomly chosen from the HapMap and for simulated SNPs with a selective advantage of 1%. These analyses used the full HapMap data, but choosing only one high- F_ST_ SNP in genomic regions where there are clusters of high- F_ST_ SNPs (see Methods). Simulations applied the *cosi* demographic model with minor modifications [7, Methods]. SNPs simulated with selection were included if there was a frequency difference >90% between ASN and YRI and where the derived allele is at high frequency in ASN. Density curves were obtained using the default settings of the density function in R [95].

We next turn to our results on SNPs that have high F_ST_ between continental groups (Figures 5 and 6). Most notably, we observed that the total number of nearly fixed differences is surprisingly low, especially at nonsynonymous sites; that there is a strong fixation bias towards non-Africans, and east Asians in particular; and that high-frequency, high- F_ST_ SNPs in east Asians generally appear to be old. However, the enrichment of genic SNPs among those SNPs with the highest F_ST_ argues against a mostly-neutral model.

A key issue for interpreting these data is the long-term rate of gene flow among continental populations. Recent population genetic studies have disagreed on whether there has been measurable gene flow between African and non-African populations [71],[74]. In principle, high rates of gene flow could prevent favored alleles from achieving high F_ST_ , and indeed, asymmetric gene flow of beneficial alleles from Africa towards east Asia could help generate the bias that we saw towards high- F_ST_ SNPs in east Asia (Figure 5). However, some aspects of the data suggest that selected alleles have generally not been able to spread freely between continental groups, and especially between Africa and east Asia (Figure 3, Supplementary Figure 15 in Text S1 and [21]). This does not rule out the possibility that selected alleles may be introduced at low frequencies by migration between broad geographic regions. A potential example of this is the light-skin allele at *SLC24A5*, which is at very low frequency in sub-Saharan Africa and east Asia (Figure 4B). However, the fact that most of the HGDP SNPs in Figure 3 are tags rather than the actual selected alleles prevents us from knowing how common it is for selected alleles to spread to low frequencies in other continents. Moreover, even if migration levels have been nontrivial, both the Asian XP-EHH results (Figure 6) and the similarity between Eurasians and all the American populations (Figures 3A, 3B) argue that there have been very few rapid, recent fixations in Eurasia.

**Figure 7:**
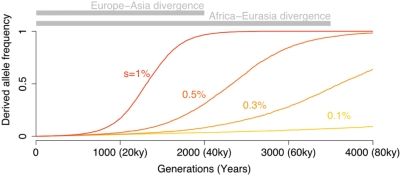
Average allele frequency trajectories of selected alleles, as a function of the strength of selection. The lines plot the mean trajectories of codominant alleles, starting from frequency 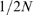 at time 0, conditional on the alleles not being lost within 4000 generations. Simulations were performed under an effective population size of 24,000 chosen to match the effective population size of the ‘Yoruba’ in *cosi* [7]. To provide some context, the bars at the top indicate the divergence times of the HapMap Europeans and Asians, and HapMap Africans and non-Africans according to the *cosi* model [7], though it should be noted that there is considerable uncertainty in the true split times. The numbers in parentheses indicate times in years, assuming 20 years per generation.

We interpret these results to imply that it is rare for strong selection to drive new mutations rapidly to near fixation. The genomic regions around high- F_ST_ SNPs in east Asians show only a modest increase in haplotype homozygosity compared to random SNPs (Figure 6). Moreover, the overall dearth of high- F_ST_ SNPs shows that strong selection has rarely acted to create nearly fixed differences between populations. The Yoruba have especially low rates of completed sweeps: for example, the HapMap data include just one nonsynonymous SNP for which the derived allele is at high frequency in Yoruba and has a frequency difference from east Asians that exceeds 90%. Figure 7 shows that the separation times between populations would have allowed ample time for strongly selected variants to fix within populations. For example, new variants with a 1% advantage could have fixed since the European-east Asian split, and variants with a 0.5% advantage could have fixed since the split of Africans and non-Africans.

Taken together, these results suggest that it is rare for variants to experience selection that is both strong enough and sustained consistently over the 10–50 KY required to drive a new mutation to fixation. Additionally, we suggest that some or all of the following factors may help to explain the data: non-African populations may have experienced more novel selection pressures than Africans; bottlenecks inflated the number of weakly selected alleles that have reached high frequency in non-Africans; and most selected traits are multigenic, and that this leads to a systematic weakening of selection on individual variants as these variants increase in frequency. We now discuss each of these factors in turn.

### Humans Experienced Novel Selection Pressures as They Left Africa

We observed more high-frequency high- F_ST_ SNPs in the HapMap Europeans and east Asians than in the Yoruba, consistent with a recent genome-wide scan for full sweeps that found few compelling signals in the Yoruba [35]. A plausible explanation is that humans experienced many novel selective pressures as they spread out of Africa into new habitats and cooler climates [75],[76]. Hence, there may simply have been more sustained selective pressures on non-Africans for novel phenotypes. The selective sweeps at skin pigmentation loci are likely examples of this.

While novel selection pressures outside Africa may be an important factor, this is likely not the entire story. In particular, this does not easily explain the excess of high-frequency high- F_ST_ alleles in east Asians compared to Europeans. (Greater drift of neutral alleles in east Asia is also unlikely to explain this pattern since the enrichment of genic SNPs among high- F_ST_ SNPs is similar in both populations (Figure 1A,B)). It is not obvious why there would be more sustained strong selection in east Asia than in Europe, and besides, our results suggest that most of these alleles were already at intermediate frequency prior to the European-east Asian divergence. A higher rate of gene flow of selected alleles between Europe and Africa than East Asia and Africa could potentially generate this result, although we currently have little evidence for widespread migration of selected alleles between the African and non-African populations (Supplementary Figure 15 in Text S1 and [21]).

It is also worth noting that this explanation does not imply an *absence* of positive selection in the Yoruba. Indeed, two studies of partial sweeps have actually reported more signals in YRI [16],[47]. African populations have presumably also experienced a variety of new selection pressures during the same time-period, due to the appearance of new pathogens, changes in diet, etc. While these pressures may have been less numerous or sustained than in non-Africans, there may also be reasons why we might have lower power to detect them. Given that African populations harbor more genetic variation than non-Africans, it is possible that there have been more sweeps on standing variation, which we are more likely to miss. Similarly, the response to selection pressures within Africa might also have been more polygenic (see below), resulting in smaller changes in allele frequencies at larger numbers of loci.

### The Interaction between Drift and Weak Selection

**Figure 8:**
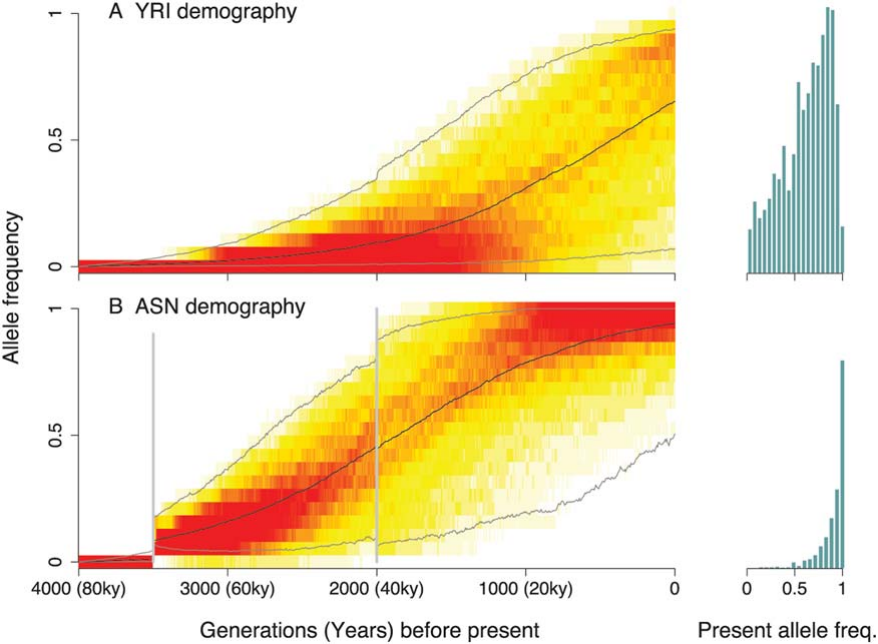
Population bottlenecks can simultaneously increase both the rate of loss and the rate of fixation of favored alleles. Trajectories of favored variants were simulated according to demographic models for the (A) Yoruba, and (B) East Asian populations [7]. In each simulation the selected variant was introduced 4000 generations before the present (∼80 KYA), i.e., prior to the out-of-Africa event. The plots show heat maps of the distributions of frequencies at each time, conditional on the allele not being lost by the present day (time = 0). The timing of bottleneck events in the model are indicated by vertical grey bars in the ASN population. Redder shades indicate a higher density of selected mutations in a particular frequency bin. The black lines indicate the mean frequencies and the grey lines bracket the central 95% of the frequency distributions. The histograms on the right show the frequency spectrum of favored mutations in the present day, for each population, excluding mutations at frequency 0. The area of each histogram is proportional to the fraction of selected alleles that have frequency >0 in the present. Notice from the histograms that a much larger fraction of favored alleles survive to the present under the YRI demography, even though the fraction of alleles that are near fixation is much smaller in the YRI.

Another important part of the explanation may be the impact of genetic drift on weakly selected variants. If strong selection is rare, then perhaps adaptation is more often due to selection on alleles with smaller fitness advantages. For selection coefficients of about 0.3% or less, the average time to fixation of a new favored allele is considerably longer than the ∼70,000 years since the split of the African and non-African HapMap populations (Figure 7). Therefore, such mutations would usually not generate extreme frequency differences between modern populations. However, since the frequency trajectory taken by a favored allele as it goes to fixation is stochastic–due to genetic drift–there will be some alleles that increase in frequency *faster* than expected. Given that the magnitude of drift since the HapMap populations diverged has been greatest in the east Asians, and least in the Yoruba, this model predicts a larger fraction of high- F_ST_ high-frequency derived alleles in the east Asians and Europeans than in the Yoruba (Figure 8 and Supplementary Figure 24 in Text S1). This greater fixation rate comes at the expense of these populations also having lost many favored alleles during bottlenecks.

While our simulations do show an east Asian fixation bias, the magnitude of the bias is smaller in the simulations than in the real data (Supplementary Figure 24 in Text S1). Hence it is possible that the effect of increased drift combines with geographic differences in selection pressures (e.g., between African and non-African environments) to generate the observed bias. Additionally, inaccuracies in the assumed demographic model might lead us to underestimate the importance of drift in east Asians. For example, it has been proposed that drift is especially active at the front of range expansions [56], [77]–[79], which might model human history better than the bottleneck model used here.

### Fluctuating Environments and Polygenic Adaptation

Additionally, properties of selection pressures themselves may contribute to the observed low rate of rapid fixation events (and small number of high- F_ST_ signals). First, it is likely that selection pressures fluctuate through time [80], and also that human cultural change modifies selection pressures through time. Thus, mutations may be driven to intermediate frequency by strong selection, but subsequently drift to loss or fixation when the selective pressure weakens.

Second, the genetic architecture of selected phenotypes has fundamental implications for the action of selection. While the genetic basis of some selected phenotypes may be monogenic (e.g. *lactase* within Europe), it is likely that most selected phenotypes are influenced by mutations at multiple genes (as seen for skin pigmentation, for example). If favored mutations increase in frequency at several genes simultaneously, then this can shift the phenotype of typical individuals of a quantitative trait towards an adaptive optimum, thus reducing the overall strength of selection on each favored mutation [81],[82]. This is a form of epistasis on fitness. Consequently, even a strongly selected phenotype may not lead to rapid fixation of favored mutations. Instead, favored mutations may increase in frequency rapidly at first, and then start to drift as the strength of selection becomes weaker.

Similarly, the “soft sweep” model in which multiple equivalent mutations sweep up simultaneously at a single locus also does not lead to full sweeps. The population adapts to a new selection pressure, but none of the favored mutations sweeps up to very high frequency [83].

### Conclusions

We have argued here that strong, sustained selection that drives alleles from low frequency to near fixation has been relatively rare during the past ∼70 KY of human evolution. Is this conclusion compatible with recent work on haplotype-based signals reporting an abundance of partial sweeps with selection coefficients of ≥1% [16],[29],[35],[47]? One possible explanation for the apparent discrepancy is that there might be many more partial sweeps than completed sweeps. This could occur if selection pressures tend to be highly variable so that favored alleles often rise to intermediate frequency and then start to drift as a result of fluctuating selection pressures or polygenic adaptation.

Alternatively, it is possible that recent studies have substantially overestimated the number and strength of partial sweeps. Perhaps the most important current challenge in selection studies is to obtain better estimates of the fraction of true positive selection signals in different types of analyses. This is especially pressing since we have shown that even extreme signals of the data have patterns that are predictable from neutral loci.

Moreover, one important unknown is the extent and strength of background selection. If background selection is concentrated in and around genes, thereby increasing the rate of drift in genic regions, it could well contribute to the observed enrichment of high- F_ST_ SNPs in genic regions [57, Supplementary Figure 4 in Text S1]. The impact of background selection for plausible biological parameters requires further investigation; see [37] for discussion of selected sweeps and background selection. If background selection is an important factor, then the role of positive selection in generating nearly fixed differences may be yet smaller than we have estimated here.

To some extent, our understanding of these issues has been hampered by the limitations and caveats of analyzing SNP data. Hopefully the next generation of genome sequence data will allow major progress on these issues. Additionally, the increasing number of genotype-phenotype associations offer the possibility of linking more selection signals to phenotypes; this may strengthen the evidence that individual signals are real and give us deeper insight into the overall impact of selection.

Finally, since high- F_ST_ SNPs are rare in the human genome, our study raises the question of whether human populations can effectively adapt to new environments or new selective pressures over time-scales of, say, ten thousand years or so. Our results seem to suggest that rapid adaptation generally does not occur by (nearly) complete sweeps at single loci. If human populations can adapt quickly to new environments, then we propose that this might instead occur by partial sweeps simultaneously at many loci.

## Materials and Methods

### HGDP Data

The HGDP consists of 1048 individuals, some of whom were previously found to be related [84]. For the analysis in this paper we used the set of 938 “unrelated” individuals genotyped previously on Illumina's “HumanHap650Y” platform [38]. The SNPs genotyped by this platform were selected to provide effective genome-wide SNP tagging in all of the HapMap populations [85].

Data cleaning and manipulation of the HGDP data was performed in PLINK [86]. We excluded 74 SNPs that were monomorphic across the entire HGDP panel, and 177 SNPs that were missing more than 5% of genotypes. To test for violations of Hardy-Weinberg Equilibrium (HWE) we constructed three large groups of individuals from three sets of populations (East Asia, Europe, Bantu Africa) that have relatively little population structure, and performed a test for HWE for each SNP within each large group [86],[87]. 1808 SNPs were removed for failing the HWE test at 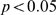 cutoff in at least two of the three groups (and have minor allele count greater than five in each group failing). We excluded 2055 SNPs in total. We note that none of the HWE-violating SNPs excluded showed pairwise population frequency differences extreme enough to contribute to Figure 2 or 3. We analyzed a total of 640,698 autosomal SNPs.

### Perlegen Dataset

Throughout the paper we make use of the Type A SNPs reported in Hinds et al. [46]. While these SNPs represent just a subset of the SNPs in HapMap Phase II, they offer two important advantages:

- The SNPs were discovered by resequencing an ethnically diverse panel of individuals from the NIH Polymorphism Discovery Panel [88], rather than single populations.
- The SNP discovery process is homogeneous over the regions resequenced. Thus the depth of coverage does not differ substantially between genomic regions covered.

The ascertainment was based on 20–50 haploid anonymous genomes isolated from the NIH Polymorphism Discovery Resource [88]. That resource is 27% European-, 27% east Asian-, 27% African-, 13% Mexican- and 13% native American [88]. The median coverage depth was 14 chromosomes per base resequenced [46]. The depth of resequencing at discovered SNPs was essentially the same for genic and non-genic SNPs. The median number of chromosomes assayed was 17 for both genic and non-genic SNPs; the mean number was 15.84 for genic and 16.17 for non-genic SNPs (personal communication, D. Hinds). This confirms that the ascertainment is indeed relatively uniform across genic and non-genic regions, suggesting that while it is an incomplete representation of all SNPs, the discovery process for Type A SNPs does not differ substantially between genic and non-genic regions due to ascertainment.

Hinds et al. [46] reported that they screened 964 MB to identify 1.62 M SNPs; they designed successful genotyping assays for 1,263,750 Type A SNPs. 896,758 of these “Type A” SNPs were genotyped in all three of the HapMap samples and have unambiguous dbSNP entries. There are a number of reasons why certain Type A SNPs were not included in the Phase 2 HapMap: the bulk of the excluded SNPs were SNPs in which it was difficult to design a genotyping assay; other criteria for exclusion included a minor allele frequency 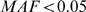 in a previous study or that SNP which is a perfect proxy (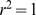) had already been typed in the HapMap [36]. None of these criteria suggest a bias in favour of preferentially including high F_ST_ SNPs in genes. Further none of the criteria should have reduced our ability to detect high F_ST_ SNPs, or bias detection towards particular HapMap populations. The MAF cutoff should not have excluded high F_ST_ Perlegen type A SNPs as they would have a global MAF well above 0.05 in [46]. While not typing perfect proxies could have excluded Perlegen SNPs from the Hapmap, a perfect proxy would still be in HapMap.

The approximate expected number of SNPs from sequencing L base pairs in 14 chromosomes would be 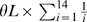, where 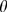 is the population scaled mutation rate per base pair (∼0.0008 in humans). This suggests that the ∼900,000 Perlegen Type A SNPs typed in HapMap represent a screen of around 345 Mb, or ∼10% of the genome (taking the genome length = 3300 Mb). We analyzed frequencies in the HapMap data, rather than in the Perlegen data, since the HapMap sample sizes are larger and Perlegen used African-Americans, who have substantial European ancestry. We used allele frequencies calculated from the HapMap phased data, with the small amount of missing data filled in by imputation. To confirm that the anonymous chromosomes in Hinds et al. [46] resequencing panel contained representatives of all three continental groups we examined the HapMap “type A” dataset for alleles present in only one of the populations and found ∼93,000 YRI-, ∼24,000 CEU-, and ∼12,000 ASN-specific alleles, suggesting that all three populations had close representatives in the anonymous resequencing panel, and so fixed differences between these populations would have been detected by the resequencing. We excluded 24 SNPs that have high F_ST_ in HapMap, but where the high F_ST_ appears to be due to allele labeling problems (allele-flips) since the reported allele frequencies in the corresponding HapMap and Perlegen samples differed by >50%.

### HapMap Data

The genotyped SNPs were identified from a variety of sources [26],[36]. Phase II includes nearly all SNPs in dbSNP release 122 that could be genotyped on the Perlegen platform [36].

To identify all non-synonymous SNPs with high levels of differentiation between HapMap populations, we used the March 2008 ‘all’ dataset from hapmap.org, consisting of 3.9 M SNPs in ASN and 3.8 M in CEU and YRI. This set contains SNPs that may have only been successfully typed in one or two populations. The list of non-synonymous SNPs with >90% frequency difference was checked by hand for potential allele calling flips using the dbSNP database and HGDP data (when the SNP was typed on this panel). A list of these non-synonymous SNPs is given in Supplementary Table 5 in Text S1.

The XP-EHH statistic was calculated on the HapMap “consensus” phased data released in July 2006 from hapmap.org, which contains all SNPs successfully genotyped in all three populations. After removing monomorphic SNPs, these data consist of 3,106,757 SNPs.

### Identification of Likely Allele Flips in the HapMap Data

We checked the highly differentiated SNPs found in consensus HapMap data for allele flips (these data are used in the main paper to identify regions for the XP-EHH analysis and in the Text S1 for XP-EHH and versions of Figure 5). We downloaded the HapMap “2007-3 redundant genotype frequencies” data, which contains information about SNPs typed by multiple centers. SNPs that had been typed by multiple centers were discarded if the centers disagreed by more than 50% in the estimate of the allele frequency in any of the three populations.

### Obtaining Genic and Ancestral States

Gene annotation information was obtained from the RefSeq database [89]. This information was primarily used for obtaining the gene start and gene end coordinates. Where required, genome coordinates were converted from NCBI build 36 (hg18) to build 35 (hg17) using the Batch Coordinate Conversion tool available at UCSC web browser [90]. A SNP was defined as nongenic if it is more than 2 kb from an annotated gene transcript; otherwise it was considered genic. Ancestral states for all SNPs were estimated using whole genome human-chimpanzee alignments from the UCSC database [90]. Based on the physical position of the SNP in the human genome (Build hg17), the allele at the corresponding position in the chimp genome (Build pantro2) was obtained. If the human SNP position aligned to missing data in the chimpanzee genome, or if the chimpanzee allele did not match either human allele, then the corresponding SNP was excluded from further analysis.

### Calculation of F_ST_

F_ST_ was calculated using the Weir and Cockerham estimator [91]. This estimator is unbiased by sample size; however, extreme values of the distribution still depend on sample size. Accordingly, we excluded low sample size populations from Figure 2.

### Clustering of SNPs with Extreme Frequency Differences

Hitchhiking results in clustering of highly differentiated SNPs, reducing the number of independent signals in the data. When we needed to ensure that independent genomic regions underlie our results or count the number of signals, we assigned strongly differentiated SNPs within 100 kb of another strongly differentiated SNP to the same cluster, such that different clusters do not contain any SNPs within 100 kb of another cluster.

### The Geographic Distributions of High- F_ST_ SNPs

To produce Figure 3, for each particular pair of comparisions (e.g. Yoruba-Han Chinese, Yoruba-French) we found all SNPs that fall in the 99.8% tail of F_ST_ for both comparisons. We then clustered these SNPs as described in ‘Clustering of SNPs with extreme frequency differences’. For each cluster we then plotted the HGDP allele frequencies for the “top” SNP for each cluster; where the top SNP was chosen by ranking SNPs in a cluster by the product of their empirical p-values in the two pairwise F_ST_ comparisons. For the HGDP Yoruba-French, Yoruba-Han comparison (Figure 3A, B) the minimum frequency difference between the pairs was 80% and 86% respectively. For the Yoruba-French, French-Han comparison (Figure 3C, D) the minimum frequency difference between the pairs was 73% and 63% respectively. For the Yoruba-Han, French-Han comparison (Figure 3E, F) the minimum frequency difference between the pairs was 79% and 63% respectively. In Supplementary Figures 10–14 in Text S1 we give versions of the plot for smaller numbers of SNPs and single pairwise comparisons. The pie chart maps were generated using the program of Wessel et al. [92].

### Haplotype Visualization

The HGDP data were phased using fastPHASE; see Text S1 for details. To visualize the haplotypes in each genomic region shown in Figure 4, we used an algorithm similar to that presented in Conrad et al. [59]. This algorithm starts by identifying the eight most common haplotypes spanning a genomic region. These eight haplotypes are called the ‘template’ haplotypes. Each template is assigned a distinct color. Next, it colors each observed haplotype as a mosaic of the eight templates, requiring exact matches between the observed haplotype and the template that is being copied. Roughly speaking, the coloring minimizes the number of switches between templates (see Text S1 for more details). Rare alleles not found on any template were dropped from the analysis in the version shown in Figure 4. The populations shown in Figure 4 are, from left to right and top to bottom: Mandenka, Russian, French, Mongola, Pima, Bantu Kenya, Druze, Balochi, Han, Maya, Biaka Pygmy, Palestinian, Makrani, Cambodian, and Papuan. For each population, 20 chromosomes were sampled without replacement for plotting.

### XP-EHH

XP-EHH was calculated as in Sabeti et al. [35]. Briefly, XP-EHH is defined relative to a given SNP 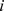 in two populations, 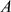 and 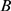. In each population, the expected haplotype homogygosity (EHH) [14] was integrated with respect to genetic distance in both directions from 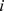. The log of the ratio of these integrals is the unnormalized XP-EHH. We chose the limit of the integration to be where the EHH in the pooled population sample 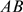 dropped below 0.05. The final XP-EHH was normalized with respect to the genome as a whole by subtracting out the mean and dividing by the standard deviation. For the analyses presented in the main text, the genetic map used was estimated by the method presented in Voight et al. [16] in the YRI population only; for the detection of selection in the ASN populations, this approach gave us the most reliable results in simulations (data not shown).

In Figure 6, XP-EHH is plotted for SNPs with a greater than 90% frequency difference between YRI and ASN. To ensure that independent signals were plotted, we clustered all SNPs with >90% frequency difference between YRI and ASN (as described in ‘Clustering of SNPs with extreme frequency differences’) and plotted the XP-EHH value for the SNP with the largest frequency difference in a cluster (choosing at random amongst tied SNPs). A version of this figure including only SNPs typed by multiple centers (to further reduce the potential for allele flips) is given in Supplementary Figure 22 in Text S1.

### Simulation Details

We used simulations that are based, with slight modifications, on a historical population genetic model, “*cosi*” [7], as this model is one of the few that incorporates both the Africa–non-Africa and Europe–east Asia population splits. This model provides a close fit to various aspects of the genetic data (Supplementary Table 4 and Supplementary Figure 25 in Text S1), although there is still considerable uncertainty about key parameters of this model, including the population split times and the amount of subsequent gene flow–if any–among them.

Simulations of haplotypes for the calculation of XP-EHH were done using a hybrid coalescent/forward-time scheme following the *cosi* model of human demography [7]. In the coalescent step, the portion of the demographic history before the split of the three populations was simulated using *cosi*. After this initialization of the population, the haplotypes were simulated forwards in time using a Wright-Fisher model. To increase efficiency, parameters were scaled by a factor of five, following Hoggart et al. [93]. That is, all population sizes and generation times were decreased by a factor of five, while all other parameters were increased by a factor of five.

As these simulations were compared to the HapMap, we had to match ascertainment and SNP density. Since the ascertainment of SNPs in the HapMap is variable and largely irreproducible, we used rejection sampling to match the joint allele frequency of the simulation SNPs and the real data [16]. We first estimated the joint allele frequency distribution of the HapMap and that of the simulations on a 12×12×12 grid of allele frequencies across the three populations. We used rejection sampling to roughly match the simulated distribution to the HapMap distribution: for each SNP in a simulation, it was accepted if a uniform(0,1) random variable 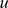 was 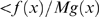, where 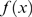 is the density in the simulations, 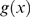 is the density in the HapMap and 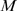 is a normalizing constant. Note that 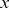 is a vector of three allele frequencies. In order to perfectly match the HapMap distribution, 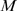 should be the maximum of the ratio between the two densities, 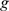 and 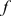. However, perfect matching to the HapMap distribution resulted in inefficient simulations; we found that a value of 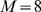 produced satisfactory results while maintaining efficiency.

Simulations of single sites (i.e. independent sites) were designed to simulate a constant rate of new mutations, 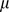 per individual per generation, with a selection coefficient 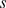. This constant rate per individual assumes that evolution is mutation limited, such that the rate of adaptation scales roughly linearly with the population size. To increase efficiency of our simulations, we modified the *cosi* demographic model [7], removing the very low levels of migration between the populations and the weak pre-out-of-Africa population expansion (both of these aspects are present in the haplotype simulations). In this model, then, there are five branches of the tree on which a new mutation can arise: the branch before the split between African and non-African populations, the branch before the split between Europe and Asia, and the three population-specific branches. For each simulation, a mutation is chosen to have arisen on a given branch 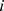 with probability 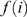; conditional on this it arises uniformly at random on this branch. The allele frequency is then simulated using a Wright-Fisher model forward in time until the present day. Alleles which are lost from the populations are discarded.

For a branch 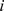, the probability that a selected allele arises on this branch, 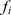, is proportional to the number of selected alleles that arise on the branch. This quantity is the time length of the branch (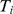) weighted by population size (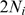) along that branch:
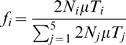
The exception is branch 1 that represents the ancestral population before the out-of-Africa split, which in our modified *cosi* model represents the population at equilibrium. To avoid having to simulate the process from far enough in the past to ensure equilibrium, we sampled the process directly from the equilibrium stationary distribution. The number of selected alleles we introduced on this branch (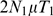), is the expectation of the number of derived selected alleles segregating at equilibrium, namely
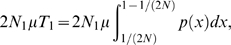
(1)where
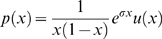
and 
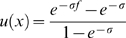
, with 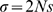 [94]. If the selected mutation is chosen to have arisen on the branch before the out-of-Africa split, we draw its allele frequency, 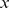, from the stationary distribution 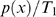 (we discretize this distribution into units of 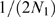).

### Statistical Analysis

We used R to perform many of the analyses and to produce most of the figures [95].

## Supporting Information

Text S1 Supplementary material.
(2.61 MB PDF)
