# Supplementary material for: The Role of Geography in Human Adaptation
Source: PLoS Genet. 2009 Jun 5;5(6):e1000500. doi: 10.1371/journal.pgen.1000500 (PMC2685456; doi:10.1371/journal.pgen.1000500)
Supplement: Text S1 — Supplementary material. (2.61 MB PDF) [file pgen.1000500.s001.pdf]

# Supplementary material for: The role of geography in human adaptation.

Graham Coop[1,2,*,†], Joseph K. Pickrell[1,*,†], Sridhar Kudaravalli[1],
John Novembre[1,3], Jun Li[4], Devin Absher[5], Richard M. Myers[5],
Luigi Luca Cavalli-Sforza[6], Marcus W. Feldman[7], and Jonathan K. Pritchard[1,8,†]

[1]Department of Human Genetics, The University of Chicago.

[2] Current Address: Department of Ecology and Evolution, University of California, Davis

[3] Current Address: Department of Ecology and Evolutionary Biology, UCLA.

[4] Department of Human Genetics, University of Michigan

[5] HudsonAlpha Institute for Biotechnology, 601 Genome Way, Huntsville, AL.

[6] Department of Genetics, Stanford University.

[7] Department of Biological Sciences, Stanford University.

[8] Howard Hughes Medical Institute.

∗ Contributed equally to this work.

† To whom correspondence should be addressed: gmcoop@ucdavis.edu,
pickrell@uchicago.edu, pritch@uchicago.edu

January 23, 2009

**Simulations of genic enrichment under positive and negative selection.** To test under what selective scenarios one expects an enrichment of large frequency differences in genic regions, we performed simulations of allele frequencies under the modified version of the *cosi* model of human demography [1] presented in the main text. We simulated 1 million independent neutral SNPs to obtain the distribution of frequency differences under neutrality, then 100K simulations each of selection coefficients of 0.0001, 0.001, and 0.003, under both positive and negative selection. We then calculated the relative density of the distributions under selection compared to that under neutrality. This is an imprecise measure of the level of genic enrichment expected in the data, as not all genic SNPs have a fixed selection coefficient (indeed, genic SNPs are likely a mixture of both neutral, hitchhiking and selected SNPs with selection coefficients of different signs and magnitudes) and not all non-genic SNPs are neutral, but our goal here was to find scenarios that match the broad overall patterns. Our results are presented in Supplementary Figure 3. The distributions of allele frequency differences under positive selection show a skew towards large values, while the distributions under negative selection show a skew towards low values. In no case did we observe an excess of large frequency differences under simulations of negative selection.

**Neutral simulations of the tail of pairwise allele frequency differences.** To better understand main text Figure 2, we performed neutral simulations to explore the relationship between mean pairwise-$F_{ST}$ and the tail of pairwise frequency differences. We used a model that was originally devised to offer a parametric estimate of $F_{ST}$ and gives a distributional form for the sample frequency of an allele in a sub-population [2]. This model has a natural interpretation under a model of gene flow and provides a null model robust to many details of demography [3]. In this model the frequency of a SNP allele in a sample has a beta-binomial distribution given the ancestral frequency of the allele, as the sample is a binomial draw from a population frequency, that itself has a beta distribution centered around the ancestral frequency $x_A$ with variance $x_A(1 - x_A)F_{ST}$. To simulate from the distribution of allele frequency differences between a pair of populations, we simulated an ancestral allele frequency from a uniform distribution. We then simulated two beta-binomial draws given this ancestral allele frequency, and calculated the difference between the sample frequencies. To estimate the upper 99.99% tail we simulated 650,000 draws from this distribution. We did this for all 321 pairwise comparisons in main text Figure 2. For each comparison we matched the mean pairwise $F_{ST}$ and sample sizes. The predicted neutral upper 99.99% tail for the 321 pairwise comparisons is shown in Supplementary Figure 7. The predicted neutral tail is lower than the observed tail, suggesting the selection has played a major role in generating the differences observed between the populations. There is also similar noise in the real and simulated data suggesting that sampling noise is likely sufficient to explain the spread of points in Main Text Figure 2. We also re-ran these simulations using an ancestral frequency distribution matched to that observed in a population which is likely a good proxy to the ascertainment population (the French HGDP population), and observed no qualitative difference. This suggests that this result is reasonably robust to ascertainment (results not shown). We chose not to simulate the maximum frequency difference under this neutral model as the value of a maximum draw is dependent on the number of independent draws from the distribution. Given that real SNPs may be linked it is difficult to model the

distribution of the maximum.

This simulation model [3] gives us reasonable support for the role of selection in generating the tails of allele frequency differentiation, but we stress that the robustness of such an extreme simulated tail to violations of the demographic model is as yet unknown. To further investigate the claim that selection generated a significant fraction of the tail of allele of allele frequency differentiation we also took a more empirical approach described in the next two sections.

**Enrichment of genic SNPs in the tails of pairwise comparisons in the HGDP data.** To show that the SNPs with extreme frequency differences between pairs of populations (shown in the main text Figure 2 and Supplementary Figure 5) are enriched for selection signals, we compared the tail of extreme frequency differentiation between pairs of HGDP populations for genic and nongenic SNPs. The two tails show a comparable level of differentiation (Supplementary Figure 8), but the genic tail is usually more extreme, with 211 out of 327 pairwise comparisons having a higher genic tail of pairwise allele frequency differentiation than the nongenic tail; this is despite the two classes showing little difference in mean frequency difference.

Differential ascertainment of genic and nongenic SNPs on the Illumina chip could either strengthen or weaken this enrichment. Further, increased sampling noise due to the smaller sample sizes in the HGDP is liable to weaken any underlying signal of enrichment. To understand these two effects we turned to the HapMap data. The subset of SNPs present on the Illumina chip has a lower 99.99% tail of differentiation in the HapMap data than does the Perlegen type-A subset of HapMap data (see Supplementary Table 2), but the tail of SNPs on the Illumina chip is similar to the tail for all HapMap SNPs. This suggests that the ascertainment of the Illumina data is missing a subset of more highly differentiated SNPs found in the more diverse ascertainment panel used by Perlegen, but is likely to be representative of the HapMap overall. The difference between genic and non-genic tails in the HGDP HapMap proxies is less pronounced than for the same subset of SNPs in the HapMap, suggesting that the smaller sample sizes of the HGDP is somewhat reducing the signal of genic enrichment. Thus, despite the apparently adverse effect of ascertainment and smaller samples the fact that we find a signal of an enrichment of highly differentiated genic SNPs is indicative of selection generating the tails of allele frequency differences.

**SNPs with high pairwise $F_{ST}$ and skin pigmentation genes.** To evaluate whether the SNPs with extreme frequency differences between pairs of populations (shown in Main Text Figure 2 and Supplementary Figure 5) are enriched for selection signals, and whether strong candidates for selection lie on the curves shown in Main Text Figure 2, we investigated how many of our extreme pairwise $F_{ST}$ SNPs fall close to pigmentation genes. Specifically we looked at how often the maximum $F_{ST}$ SNP in a pairwise comparison (or one of the $\sim 65$ SNPs in the 99.99% tail) was within 50kb of a gene known from mapping studies to affect skin pigmentation (*KITLG, MC1R, OCA2, SLC24A4, SLC24A5, SLC45A2* and *TYR*). We color pairwise comparisons that meet this criterion red in Supplementary Figure 9.

To assess the probability that a SNP chosen without regard to $F_{ST}$ falls close to a pigmentation gene, we randomly sampled 50,000 autosomal SNPs on the Illumina panel; we find

that only 25 of these SNPs fall near to the pigmentation genes (i.e., at a rate of $5 \times 10^{-4}$). Thus, under the null that pigmentation genes are not enriched in the class of SNPs with the maximum autosomal $F_{ST}$ in pairwise comparisons, the top panel of Supplementary Figure 9 (with 327 points) should have less than 1 red point. Assuming that the 65 SNPs in the 99.99% pairwise $F_{ST}$ tail are independent the probability that at least 1 falls near a pigmentation gene is 3% $(1-(1-5\times10^{-4})^{65})$. Thus if the high $F_{ST}$ SNPs were unassociated with skin pigmentation genes only $\sim 9$ of the points should be red in the lower panel of Figure 9. Clearly both panels have many more SNPs close to skin pigmentation genes than expected by chance.

**Phasing.** The genotypes from the HGDP data were phased using fastPHASE v1.2 [4]. Extensive testing of the accuracy of various approaches for phasing these types of data were presented in Conrad et al. [5]; we closely follow their approach. Briefly, we phased all the individuals together using the fastPHASE model which allows variation in the switch parameter across subpopulations. We used seven subpopulations, corresponding to the populations obtained from clustering at neutral loci. The HapMap YRI and CEU haplotypes were included as haplotypes with known phase (as they were obtained from trio data and are highly accurate), and the HapMap ASN genotypes were also included in the phasing.

**Haplotype Visualization.** To visualize world-wide haplotypes in a region, we use the algorithm developed by Conrad et al. [5]. We start by identifying the eight most common haplotypes worldwide that span a genomic region. These eight haplotypes will be called the 'template' haplotypes. Next we color each observed haplotype as a mosaic of the eight templates. We start in the physical center of the genomic region, and identify the largest segment that exactly matches one template. That segment is colored according to the color of the template. Next, we move immediately to the right of the colored segment, and color the largest possible segment that exactly matches one of the templates and that has a left-hand edge at the right edge of the region that has already been colored. This process is continued until the right-hand end of the genomic region is reached. An analogous process is then performed to the left of the central block. Note that sometimes a rare allele is not found on any template. We ignore these rare alleles when creating the mosaic structure. For clarity, the plotted chromosomes are sorted by the coloring in the center of the region.

**Geographic sharing of partial sweeps.** To investigate the sharing of partial sweep signals between geographical regions, we examined the overlap between significant iHS signals among the 7 broad geographical regions of the HGDP. The geographic regions are the Bantu Africans, Europe, Middle East, South Asia, East Asia, Oceania and the Americas. Within Africa we use only the Bantu Africans–i.e. excluding the Pygmy and San populations–because the deep structure within Africa could create false high iHS signals and reduce power to detect real signals. We calculated the iHS statistic separately within each of these 7 broad geographic regions for all Illumina SNPs with minor allele frequency $> 5\%$ [6]. To calculate iHS for a SNP we calculate the integral of the decay of haplotype homozygosity as a function population scaled recombination rate separately for both the ancestral and derived alleles, and take the absolute value of the log of the ratio of the two integrals [see 6,

for futher details]. We then divided up the genome into non-overlapping windows of 200kb each. For each broad geographical region each window was given an empirical rank by how many SNPs within that window had an $|iHS| > 2$ (corresponding to being in the upper 95% empirical tail of $|iHS|$ SNP scores). For the top 1% ranked windows for a particular broad geographical region, we then determined what other broad geographical regions ranked this genomic window in their 5% empirical tail. For each of the top ranked windows in a broad geographic region there is a set of other broad geographic regions that the window also is significant in (for example a window significant at the 1% level in Europe may also be significant at the 5% level of iHS in the Middle East and South Asia, but not significant in other comparisons). To illustrate the sharing of iHS signals for each broad geographic region we took the four most common combinations of other broad geographic regions and show their counts in Supplementary Figure 15.

**Ancestral alleles hitchhiking with the selected variants.** Throughout the paper we use the derived allele at SNPs with high differentiated allele frequencies to determine in which population the putative selected sweep occurred in. This can be confounded if the ancestral (chimpanzee) allele at a SNP is mis-called (e.g. due to CpGs) or because a low frequency ancestral allele has hitch-hiked with the selected allele. To investigate how often hitchhiking ancestral alleles lead to large frequency differences between populations, and hence misclassification of where the selected sweep occurred, we first performed simulations using our modified version of the *cosi* model [1]. Looking at 125 kb on either side of a recently fixed allele in ASN, for a selection coefficient of s= 1% (in 170 simulations), excluding the selected site, and in the pseudo-hapmap data, a sweep creates on average 12 large frequency differences ($|\delta| > 0.9$), 11 of which have high derived allele frequency in ASN. (For comparison, the average neutral region has 0.05 such SNPs, 90% of which are high frequency derived in ASN). Similarly in selection simulations of sweeps in YRI (in 120 simulations), a sweep creates about 17 snps with $|\delta| > 0.9$, 14 of which have high derived allele frequency in YRI.

This mis-classification issue should not have biased our conclusion that most nearly fixed differences involve derived alleles in out of Africa populations. However, the mis-classification does appear to have inflated the number of putatively selected alleles near fixation in the YRI. For example, of the 744 SNPs in figure 17 with > 90% frequency difference between ASN and YRI the small fraction that are nearly fixed derived differences in the Yoruba cluster with multiple SNPs showing the same level of differentiation but with the derived allele at high frequency outside Africa (see Supplementary figure 21). There appear to be few clusters of SNPs with large allele frequency differences, which appear derived in the YRI.

**Rate of adaptive evolution in Yoruba at nonsynonymous sites.** We observed just 1 nonsynonymous SNP in HapMap and 0 nonsynonymous SNPs in the Type A HapMap SNP set for which the derived allele is at high frequency in YRI and has a frequency difference > 90% between YRI and either CEU or ASN. Since Perlegen screened $\sim 10\%$ of the genome, and the HapMap covers rather more, we suggest that there are probably $\leq\sim 5$ such SNPs in the entire genome. Hence, in total numbers, a tiny fraction of genes in the genome have

been targets of rapid adaptive nonsynonymous fixations in Yoruba in the past ~70 KY.

Moreover, since the separation time between YRI and CEU+ASN is approximately 1% of the separation time of humans and chimpanzees, this would lead us to estimate, very roughly, about 100-500 rapid adaptive nonsynonymous fixations on the human lineage (or 200-1000 on both branches), if humans have evolved at a constant rate of adaptive evolution. This rate is considerably less than recent estimates suggesting that above 10% of the 38,000 amino acid differences between humans and chimpanzees were adaptive [7].

There are several possible explanations for the discrepancy (assuming that the 10% estimates are correct). Our favored explanation is that most adaptive fixations are relatively weak, taking longer 70,000 years to fix. It is also possible that we are understating the propensity of mutations that are favored in YRI to spread to CEU and ASN, and hence underestimate the number of fixation events in YRI. Finally, it is possible that the recent rates of adaptation in YRI are relatively low compared to the long-term average, in contrast to recent claims [8].

**Assessment of the *cosi* model of human demography.** Schaffner et al. [1] developed a model of human demography based on the HapMap. Since the publication of that model, Keinan et al. [9] assembled a list of SNPs ascertained in a uniform manner–by virtue of being heterozygous in an individual of known ancestry. This allows for a somewhat independent test of the model. To do this, we performed 1000 simulations as described in the main text with $s = 0$, and ascertained SNPs as in Keinan et al. [9]. That is, for each simulation, three sets of SNPs were constructed: one from SNPs polymorphic in a sample of two "YRI" chromosomes, one from SNPs polymorphic in a sample of two "CEU" chromosomes, and one from SNPs polymorphic in a sample of two "ASN" chromosomes. The allele frequencies for these SNPs were then reported based on the full samples (120 chromosomes in YRI and CEU, and 180 in ASN).

The data from Keinan et al. [9] allow for assessment of the performance of the *cosi* model in generating two aspects of the data–the allele frequency spectrum and population differentiation, which we measure here using Fstns.

In Figure 25, we show the allele frequency spectra from Keinan et al. [9] and that generated from our simulations under the cosi model with the same ascertainment scheme. The spectra are quite similar. There is a slight excess of low frequency polymorphisms in our simulations of the CEU and ASN populations as compared to the true data, suggesting the bottlenecks in the *cosi* model may be too modest. Overall, however, the model recapitulates the qualitative aspects of the data rather impressively.

We next looked at $F_{ST}$ . The estimated mean pairwise $F_{ST}$ between populations depends on the population in which the SNPs were ascertained; in Supplementary Table 4 we present mean $F_{ST}$ for all three pairwise comparisons for all three ascertainment panels. Overall, again we see a generally good fit.

| Frequency difference | $< -70\%$ | $> 70\%$ |
|---|---|---|
| ASN-YRI | 1113 | 5842 |
| CEU-YRI | 601 | 2899 |
| ASN-CEU | 457 | 335 |

Supplementary Table 1: **The number of Perlegen Type A SNPs with an absolute frequency difference $> 70\%$ between pairs of HapMap populations.** (See main text Figure 1). The numbers in the left and right columns give the number of SNPs where the derived allele is at high frequency in the second and first population in the pair respectively.

| Comparison | genic | non-genic |
|---|---|---|
| Perlegen | | |
| CEU-ASN | 0.83 | 0.777 |
| CEU-YRI | 0.891 | 0.867 |
| YRI-ASN | 0.953 | 0.927 |
| HGDP SNPs in HapMap | | |
| CEU-ASN | 0.776 | 0.733 |
| CEU-YRI | 0.834 | 0.825 |
| YRI-ASN | 0.909 | 0.895 |
| HGDP SNPs in HGDP | | |
| Fra-Han | 0.848 | 0.816 |
| Fra-Yor | 0.899 | 0.893 |
| Han-Yor | 0.956 | 0.947 |

Supplementary Table 2: **The 99.99% tail of the frequency difference between pairs of populations for genic and nongenic SNPs.**

| Comparison | Number of SNPs | Number of regions |
|---|---|---|
| ASN-YRI | 376 | 122 |
| CEU-YRI | 56 | 32 |
| ASN-CEU | 8 | 5 |

Supplementary Table 3: **Numbers of SNPs with $> 90\%$ frequency difference between HapMap populations and the number of genomic regions into which these SNPs cluster.** We excluded SNPs that were typed by only a single center ($\sim$ 50% of SNPs), because we would not detect allele flips in these SNPs. In comparisons between CEU-YRI and ASN-CEU we excluded a particularly high fraction of potential allele flips, presumably because there are fewer SNPs that are extreme between these regions. Thus for these two comparisons (Supplementary Figures 19 and 20) we present only SNPs that had been confirmed by multiple centers to guard against allele flips. Note that the SNPs used in Supplementary Figures 18, 19 and 20 are subsets of these, as ancestral states could not be determined for all SNPs.

| Ascertainment Panel | YRI-CEU $F_{ST}$ | YRI-ASN $F_{ST}$ | CEU-ASN $F_{ST}$ |
|---|---|---|---|
| YRI | 0.074 (0.068) | 0.083 (0.082) | 0.041 (0.049) |
| CEU | 0.071 (0.078) | 0.082 (0.088) | 0.053 (0.072) |
| ASN | 0.070 (0.071) | 0.081 (0.092) | 0.051 (0.072) |

Supplementary Table 4: **A comparison of mean $F_{ST}$ between *cosi* simulations and Keinan et al.'s [9] data.** The table shows mean Fst for each pairwise comparison from each Keinan et al. [9] ascertainment panel and (in parentheses) the corresponding values obtained from simulations in our implementation of the *cosi* model for SNPs ascertained in the same manner. See Supplementary Section 'Assessment of the *cosi* model of human demography' for more detail.

| **CEU-YRI** | | | | | |
|---|---|---|---|---|---|
| rs | YRI | CEU | ASN | potential allele flip | High derived allele frequency |
| rs608620 | 0.058 | 0.992 | NA | No information | YRI |
| rs1871534 | 0.017 | 1.000 | 1.000 | No | YRI |
| rs2289541 | 0.000 | 0.990 | 0.893 | Yes | |
| rs8131523 | 0.000 | 0.912 | 0.897 | Yes | |
| rs1426654 | 0.025 | 1.000 | 0.011 | No | CEU |
| rs16891982 | 1.000 | 0.017 | 0.994 | No | CEU |
| rs2269529 | 0.958 | 0.000 | 0.422 | Yes | |
| rs5896 | 0.000 | 0.950 | 0.000 | Yes | |
| rs2227852 | 1.000 | 0.008 | 1.000 | No Information | CEU |
| rs364637 | 0.000 | 1.000 | 0.000 | Yes | |
| rs4422842 | 1.000 | 0.000 | NA | No information | CEU |
| **ASN-YRI** | | | | | |
| rs | YRI | CEU | ASN | potential allele flip | High derived allele frequency |
| rs7720480 | 0.936 | NA | 0 | No information | ASN |
| rs17822931 | 1 | 0.879 | 0.067 | No | ASN |
| rs1044498 | 0 | 0.873 | 0.939 | No | ASN |
| rs1047626 | 0.942 | 0.267 | 0.034 | No | ASN |
| rs749670 | 1 | 0.625 | 0.08 | No information | ASN |
| rs6546839 | 0.075 | 0.8 | 0.989 | No | ASN |
| rs6546837 | 0.075 | 0.8 | 0.989 | No information | ASN |
| rs1871534 | 0.017 | 1 | 1 | No | YRI |
| rs6724782 | 0.068 | 0.8 | 0.989 | No | ASN |
| rs12075 | 0.000 | 0.483 | 0.904 | No | ASN |
| rs3911730 | 0.933 | 0.108 | 0 | No | ASN |
| rs3813227 | 0.075 | 0.8 | 0.989 | No | ASN |
| rs602990 | 0.083 | 0.475 | 0.989 | No | ASN |
| **ASN-CEU** | | | | | |
| rs | YRI | CEU | ASN | potential allele flip | High derived allele frequency |
| rs2303772 | 0.898 | 0.000 | 0.977 | Yes | |
| rs8110904 | 0.567 | 0.008 | 1.000 | Yes | |
| rs1426654 | 0.025 | 1.000 | 0.011 | No | CEU |
| rs16891982 | 1.000 | 0.017 | 0.994 | No | CEU |
| rs8044843 | 0.161 | 0.000 | 0.978 | Yes | |
| rs5896 | 0.000 | 0.950 | 0.000 | Yes | |
| rs2227852 | 1.000 | 0.008 | 1.000 | No information | CEU |
| rs364637 | 0.000 | 1.000 | 0.000 | No information | CEU |

Supplementary Table 5: **Non-synonomous SNPs in HapMap Phase II with $>90\%$ frequency difference between pairs of HapMap populations.** These SNPs were checked in dbSNP and the HGDP data by hand for potential allele flips. We list: the allele frequency in each of the 3 HapMap populations; whether the SNP appears to be an allele flip; and if not which of the pair of populations has high derived allele frequency.

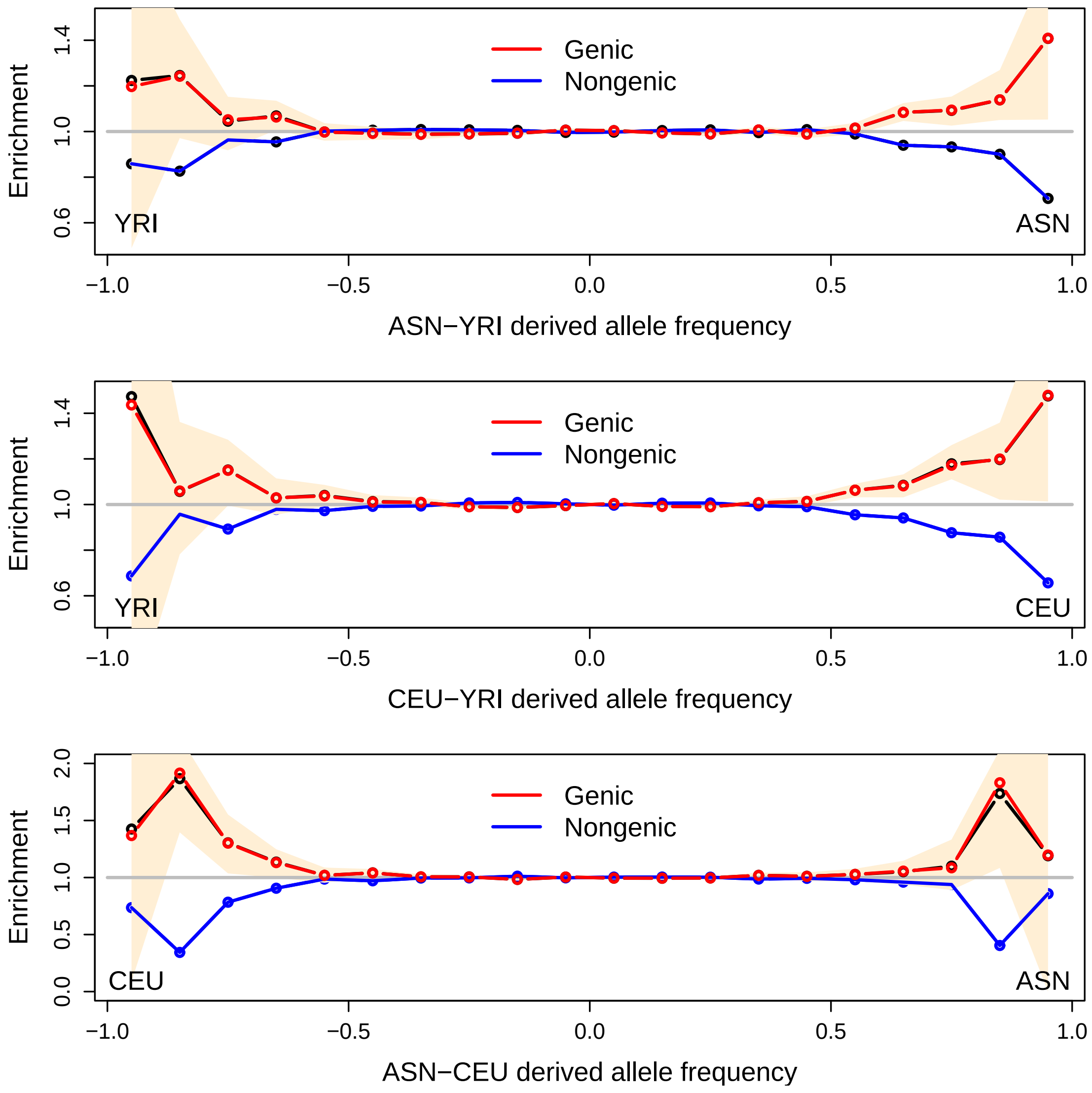


Supplementary Figure 1: **A version of main text Figure 1 with 95% confidence intervals.** See caption of main text Figure 1 for details.

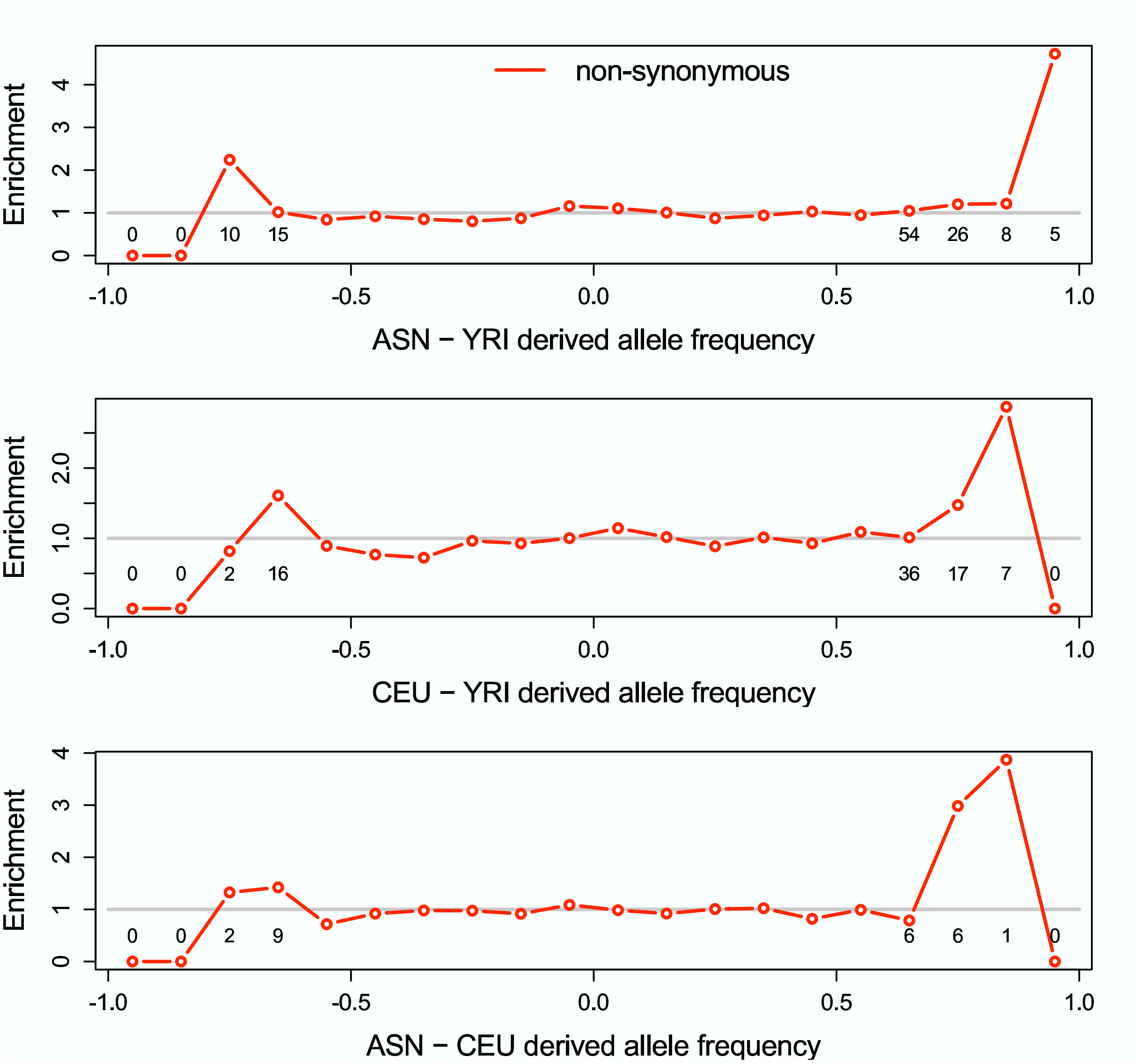


Supplementary Figure 2: **The enrichment of non-synonymous SNPs in the tails of allele frequency differentiation in the Perlegen type A SNPs.** See caption of main text Figure 1 for details. The numbers next to each point give the number of non-synonomous SNPs in that bin.

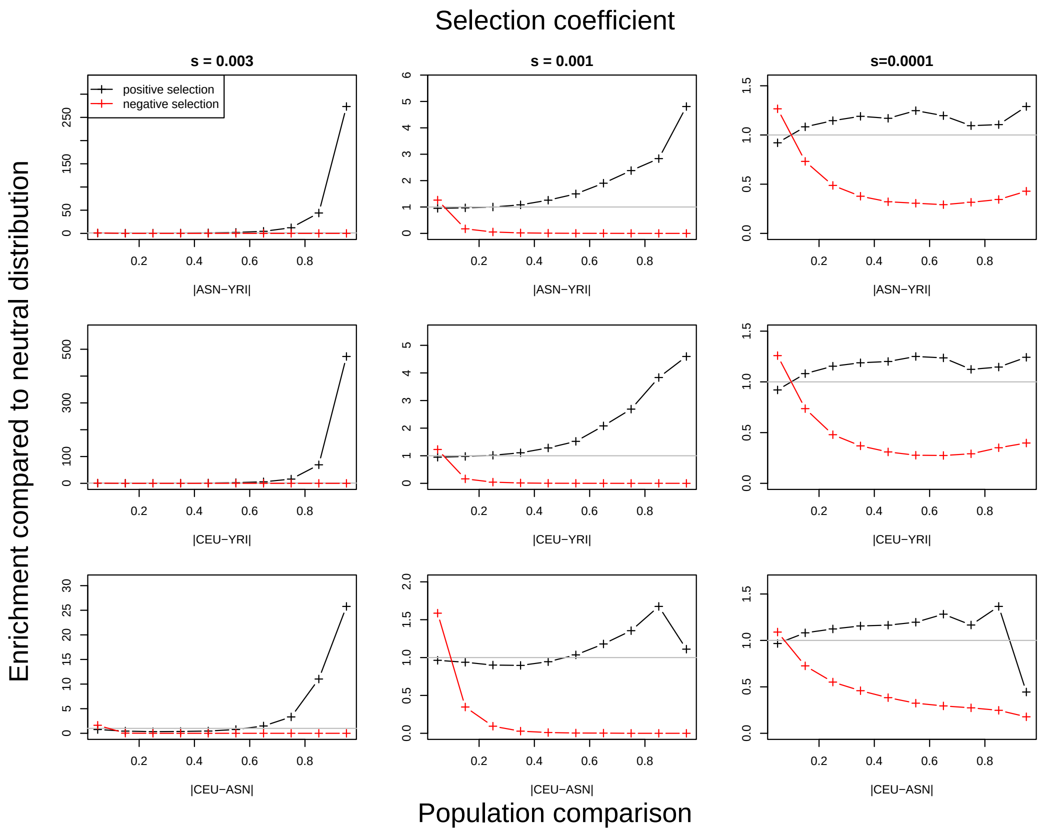


Supplementary Figure 3: **The simulated distribution of allele frequency differences under different selection coefficients.** For each selection coefficient (both positive and negative), we estimated the distribution of the absolute value of the frequency difference between all three pairwise comparisons of simulated populations (YRI=Yoruban, CEU=European, ASN=East Asian). These distributions were then binned into ten bins and compared to the distribution under neutrality. Each panel shows a given pairwise comparison for a given selection coefficient, for both positive (black) and negative (red) values of the selection coefficient. See Supplementary Section 'Simulations of genic enrichment under positive and negative selection' for details.

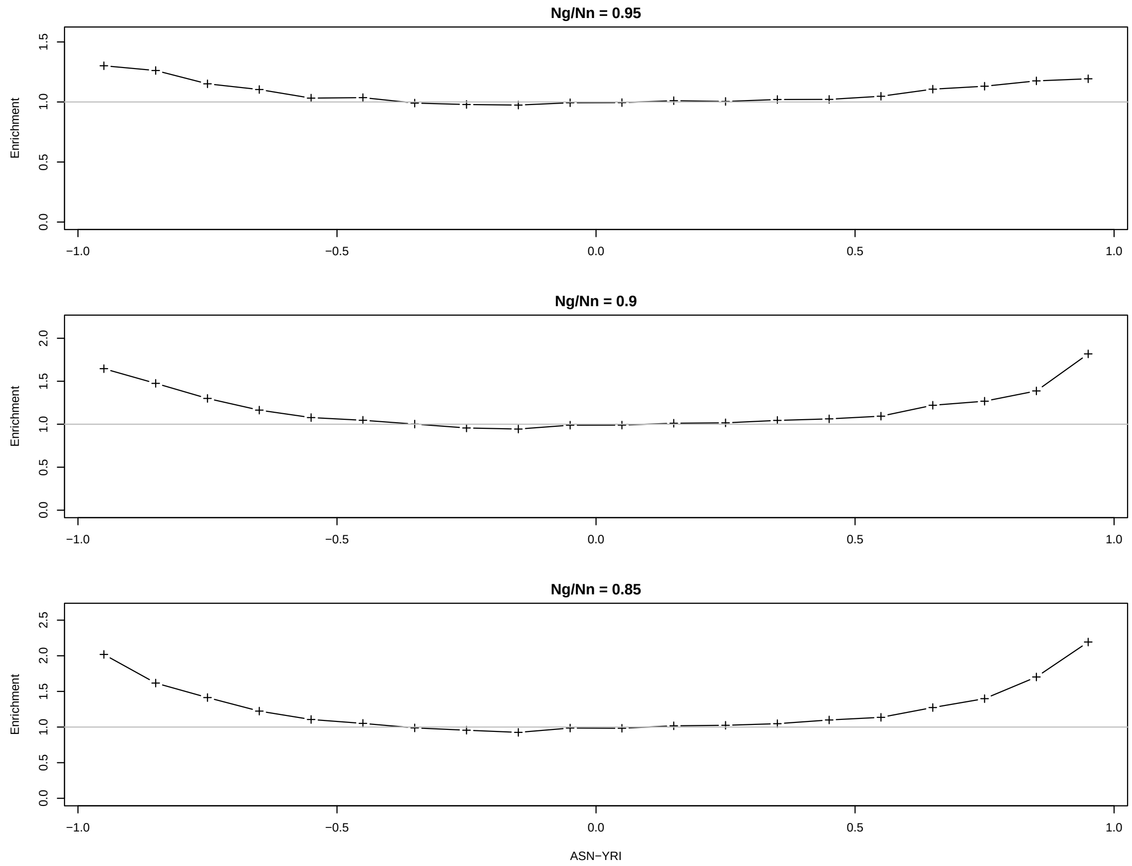


Supplementary Figure 4: **The simulated distribution of allele frequency differences under different levels of background selection.** To a first-order approximation background selection simply increases the rate of genetic drift, thus we can model background selection as a local reduction in the effective population size of a region. To simulate the effects of this, we performed simulations of single sites using the *cosi* model of human demography described in the main text. Initially, we generated the distribution of allele frequency differences between ASN and YRI under the standard *cosi* model with one million simulations, then we generated the same distribution in simulations where all the population sizes have been reduced to some fraction of the original size (in the figure are those for reductions of $N_e$ to 95%, 90%, and 85% of the standard *cosi* model). For bins of allele frequency difference, we plot the relative enrichment of SNPs under the smaller $N_e$ model (ie. background selection) to that under the neutral model.

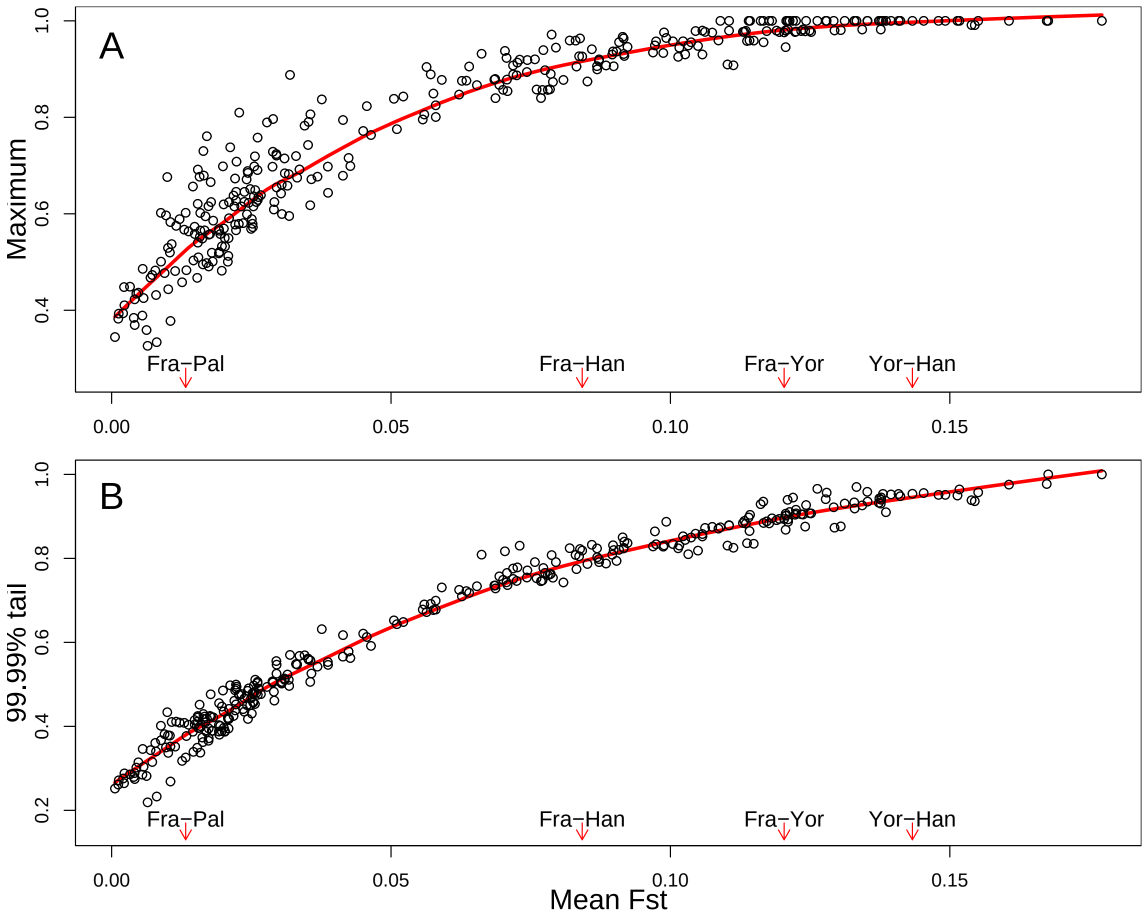


Supplementary Figure 5: **The relationship between mean $F_{ST}$ and the extremes of $F_{ST}$ for pairwise comparisons of HGDP populations.** The x-axis of each plot shows the autosomal mean $F_{ST}$ for pairs of HGDP populations, considering all possible pairs from among the 26 HGDP populations with samples $\geq$ 15 individuals. The y-axes show the value of **(A)** the maximum autosomal $F_{ST}$ for each population pair, and **(B)** the value of the 65th most extreme autosomal $F_{ST}$ for each population pair (i.e., corresponding to the 99.99th percentile of the $F_{ST}$ distribution). To provide a sense of scale on the figure, red arrows are used to indicate the mean autosomal pairwise $F_{ST}$ between some arbitrary pairs of populations (key: French (Fra), Palestinian (Pal), Han-Chinese (Han) and Yoruba (Yor)). The red line is guide to the eye fit using the lowess function in R.

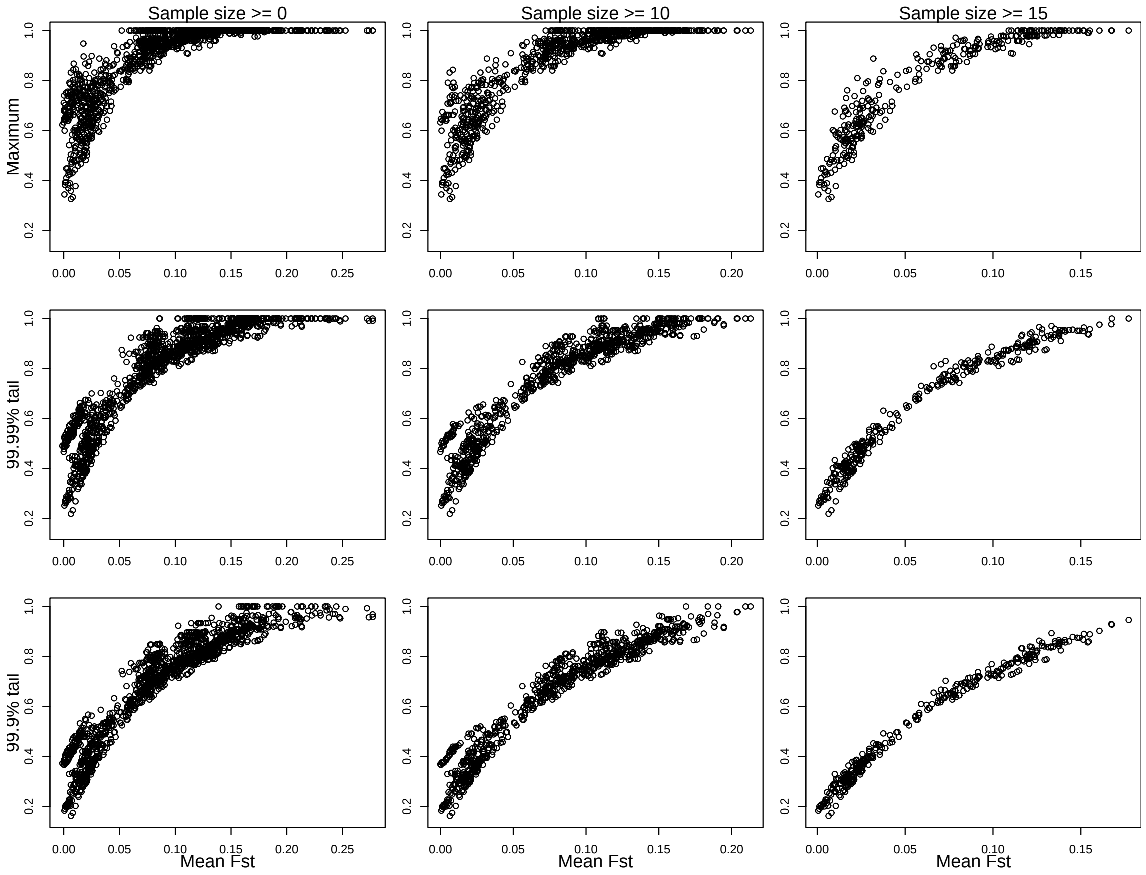


Supplementary Figure 6: **A version of Supplementary Figure 5 for different sample size cutoffs.** (Number of individuals). Smaller sample sizes lead to inflated tails of $F_{ST}$ due to increased sampling variance, and so populations with small sample size were omitted from main text Figure 2. We note that some of the more isolated HGDP populations have small sample size (e.g. the San) and so are not present in comparisons in main text Figure 2, thus larger sample sizes are needed for these groups to investigate whether any of them are outliers from the curves.

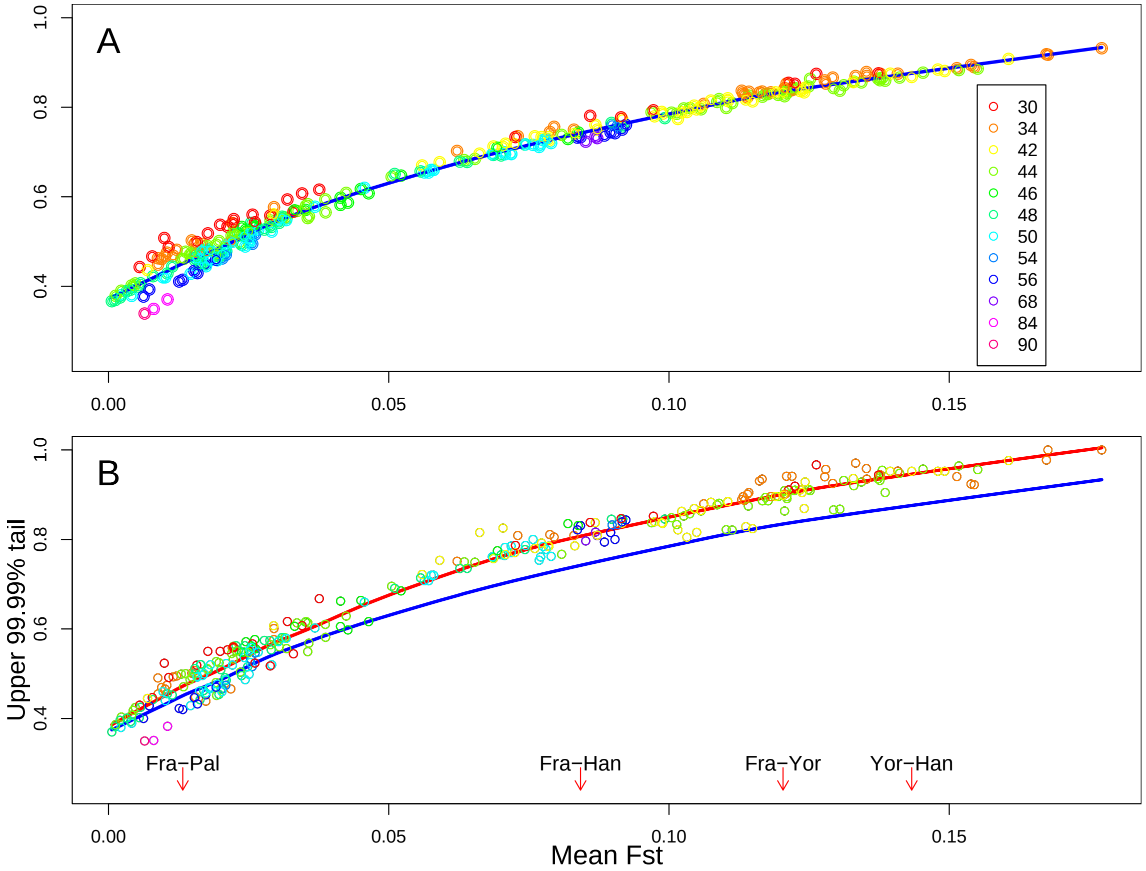


Supplementary Figure 7: **A comparison of the relationship between mean pairwise $F_{ST}$ and the tail of pairwise allele frequency differences in simulations and the HGDP data.** A) The 99.99% tail of allele differences between pairs of populations (y-axis) simulated to match the mean $F_{ST}$ (x-axis) and sample sizes observed in the HGDP data. The points are colored, as indicated by the legend, to show the smaller of the two sample sizes (number of individuals) in the comparison. B) The 99.99% tail of allele differences between pairs of HGDP populations (y-axis) plotted against the mean $F_{ST}$ (x-axis) for all populations with a sample size $\geq$15, points are colored by minimum sample size. The red line is a lowess curve fit to the observed data. The blue line in both panels is a lowess curve fit to the simulated data. See Supplementary Section 'Neutral simulations of the tail of pairwise allele frequency differences' for details.

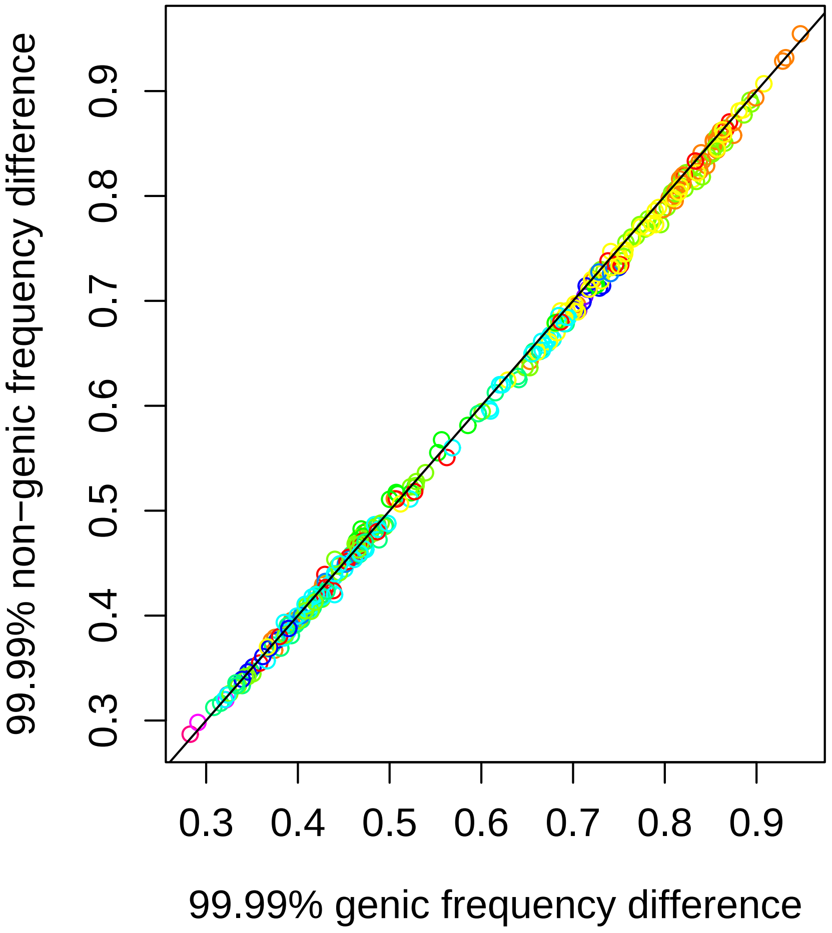


Supplementary Figure 8: **A comparison of the genic and nongenic tails of pairwise allele frequency differences across HGDP populations.** The points are colored by the minimum of the two sample sizes, as in Supplementary Figure 7. The black line gives x=y, the majority of points are under the line indicating that the tail for genic SNPs is more extreme than for nongenic SNPs. See Supplementary Section 'Neutral simulations of the tail of pairwise allele frequency differences' for details.

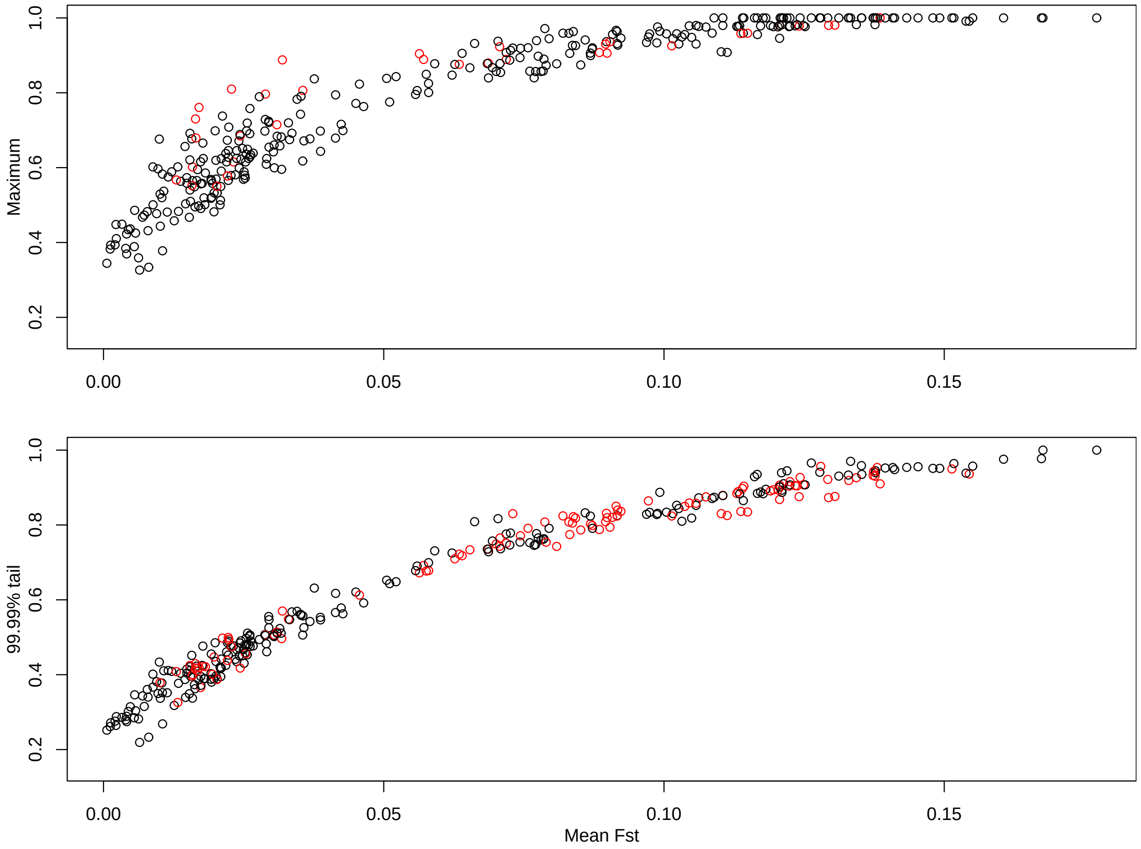


Supplementary Figure 9: **The enrichment of skin pigmentation genes close to SNPs with extreme frequency differences.** A version of Supplementary Figure 5, where pairwise comparisons in the top panel are colored red if the most extreme pairwise differentiated SNP falls within 50kb of a skin pigmentation gene, and in the lower panel they are colored red if any of the 65 most extreme pairwise differentiated SNPs falls within 50kb of a pigmentation gene. See Supplementary Section 'SNPs with high pairwise $F_{ST}$ and skin pigmentation genes' for more details.

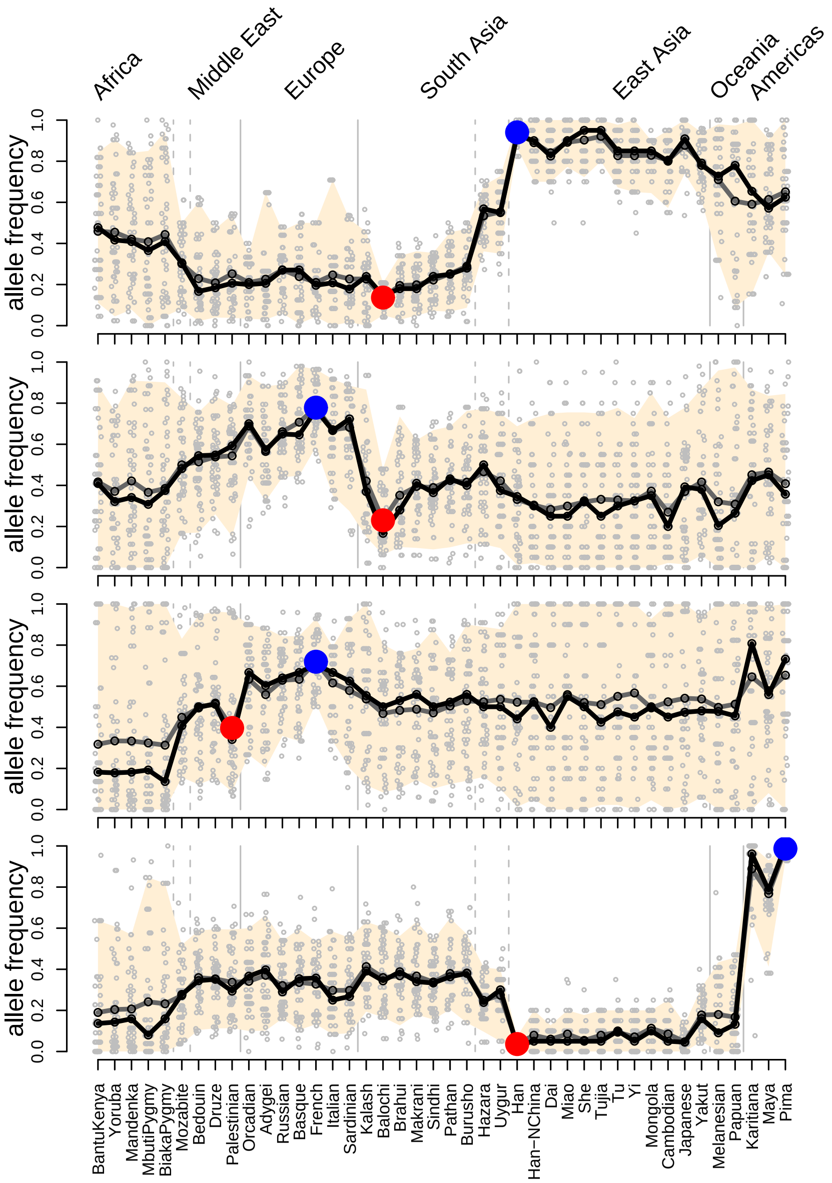


Supplementary Figure 10: **Global allele frequency distributions at SNPs that are highly differentiated between other pairs of HGDP populations** (indicated by red and blue dots). The rows correspond to SNPs with extreme frequency differences between Balochi-Han, French-Balochi, Palestinian-French, Han-Pima. Each plot shows 50 SNPs with extreme $F_{ST}$ between pairs of populations (representing 50 independent genomic regions in each plot; see Methods). See the caption of main text Figure 3 for more detail. The allele frequency at each SNP is polarized so that the major allele in the population marked by the blue dot is plotted.

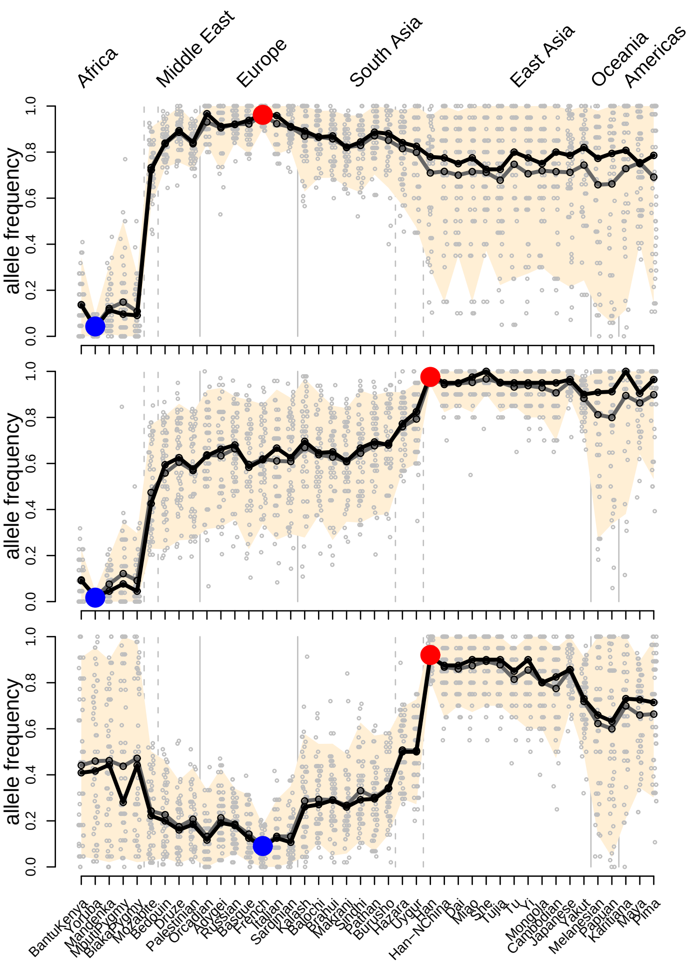


Supplementary Figure 11: **Global allele frequency distributions at SNPs that are highly differentiated between pairs of HGDP populations** (indicated by red and blue dots). The rows correspond to SNPs with extreme frequency differences between Yoruba-French, Yoruba-Han, and Han-French. Each plot shows 50 SNPs with extreme $F_{ST}$ between pairs of populations (representing 50 independent genomic regions in each plot; see Methods). See the caption of main text Figure 3 for more detail. The allele frequency at each SNP is polarized so that the minor allele in the population marked by the blue dot is plotted.

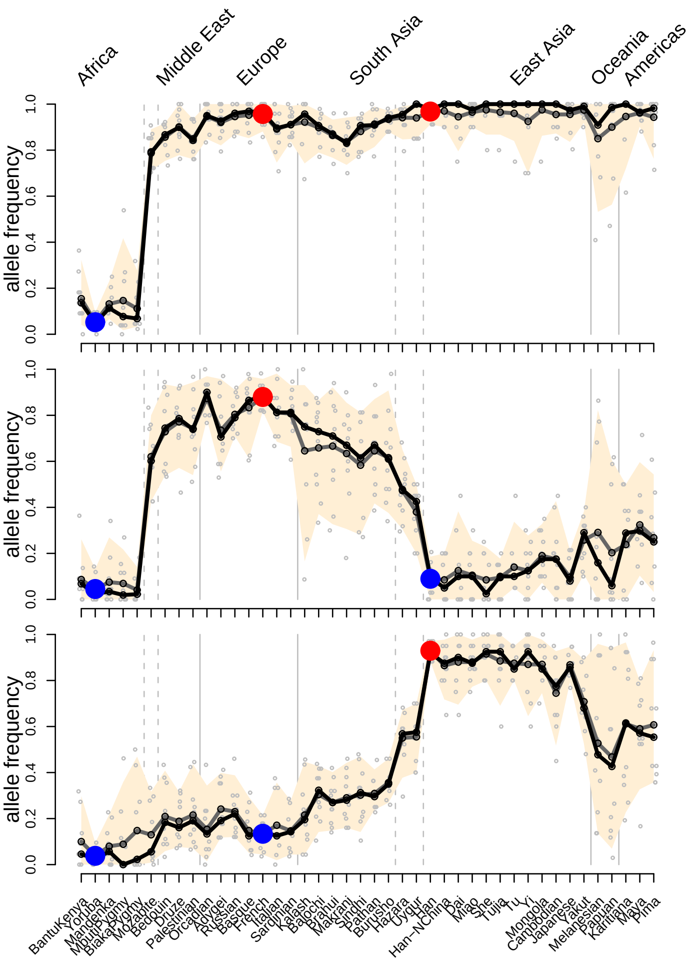


Supplementary Figure 12: **A version of the main text Figure 3 where each plot shows 10 SNPs** with extreme $F_{ST}$ between pairs of populations (representing 10 independent genomic regions in each plot; see Methods). See the caption of main text Figure 3 for more detail.

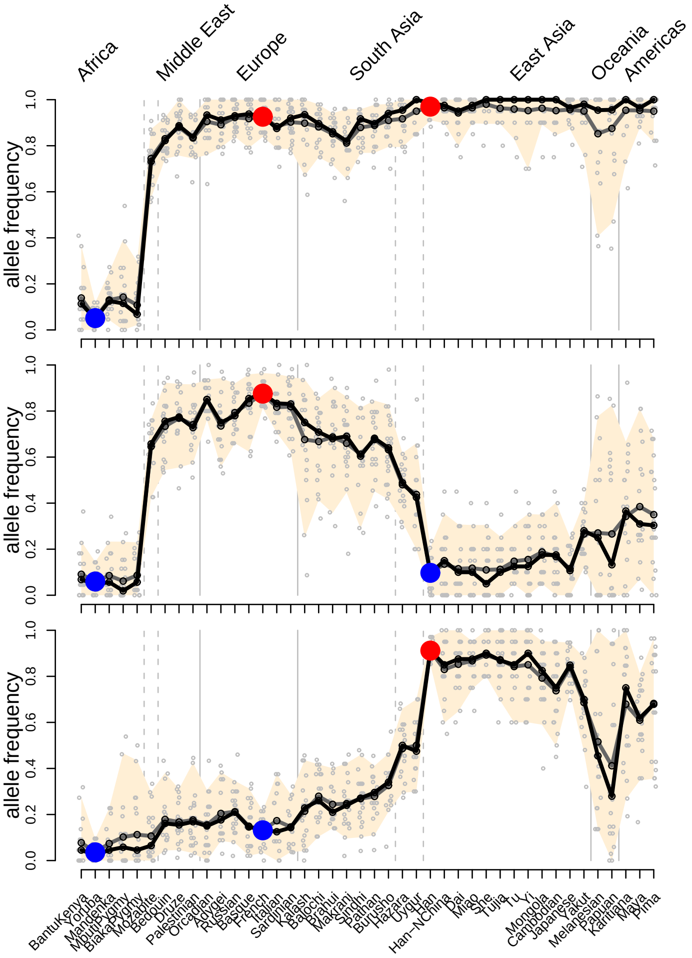


Supplementary Figure 13: **A version of the main text Figure 3 where each plot shows 20 SNPs** with extreme $F_{ST}$ between pairs of populations (representing 20 independent genomic regions in each plot; see Methods). See the caption of main text Figure 3 for more detail.

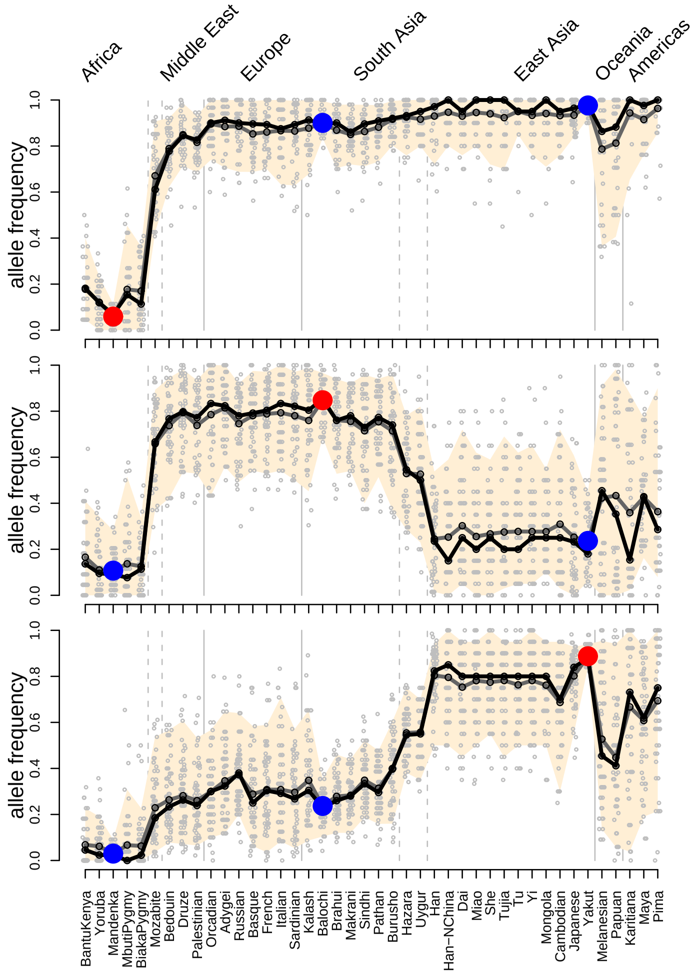


Supplementary Figure 14: **A version of the main text Figure 3, for a different choice of populations, where each plot shows 50 SNPs** with extreme $F_{ST}$ between pairs of populations. Each row plots frequency distributions for 50 of the most extreme SNPs genome-wide in the following pairs of comparisons: **(A)**: SNPs for which Mandenka is highly differentiated from both Balochi and Yakut; **(B)**: Balochi is differentiated from Yakut and Mandenka; **(C)**: Yakut is differentiated from Balochi and Mandenka. See the caption of main text Figure 3 for more detail. SNPs were polarized to plot the minor allele in the Mandenka.

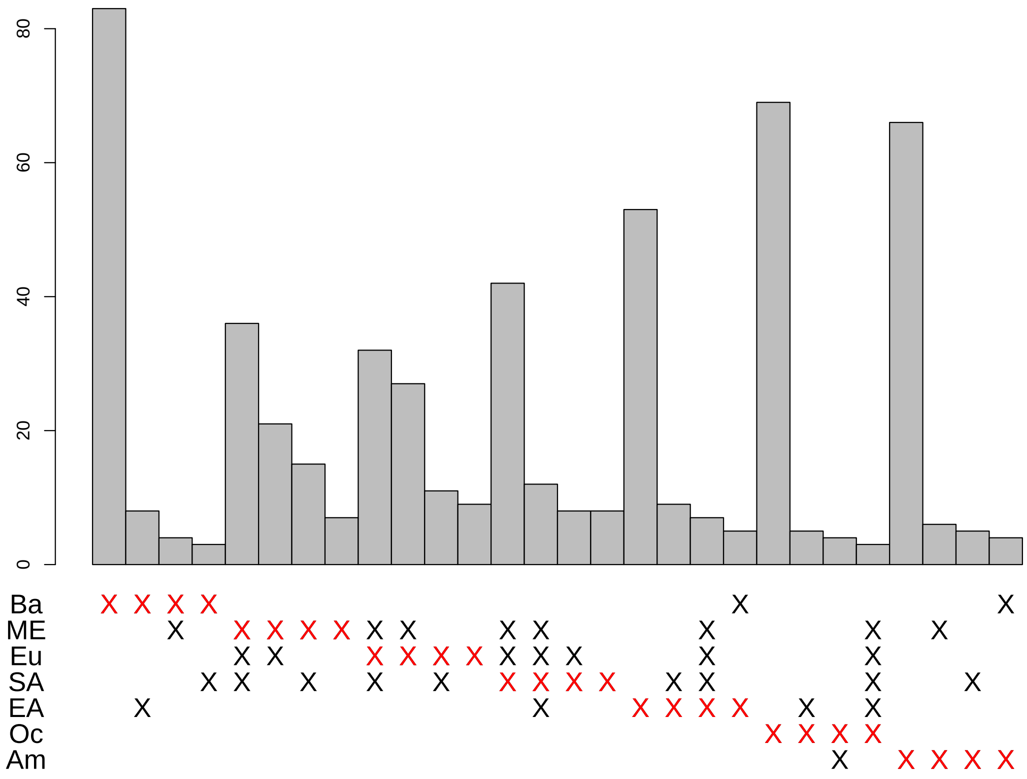


Supplementary Figure 15: **Sharing of partial sweep signals among geographic regions.** (Ba:Bantu, ME:Middle East, Eu:European, SA: south Asian, EA: east Asia, Oc:Oceania and Am:American). The bar chart indicates sharing of iHS signals across these broad geographic regions. For each geographic region in turn, we considered all genomic windows for which iHS was in the top 1% of the empirical distribution (red crosses). We then determined for which other geographic regions this window was in the top 5% and indicate the frequencies of the four most common combinations of sharing across regions by the vertical bar and black crosses. So for example, when we consider genomic regions in the 1% tail for Bantu (red crosses in the Bantu row), there are $2^6$ other possible combinations of signals present/absent across the other six populations. The vertical columns of crosses indicate the four most frequent combinations that occurred among these $2^6$ possibilities: i.e., Bantu only, Bantu and east Asia, Bantu and middle East, Bantu and south Asia. The heights of the vertical bars indicate the numbers of genomic regions that exhibit each such pattern. See Supplementary Section 'Geographic sharing of partial sweeps' for more detail.

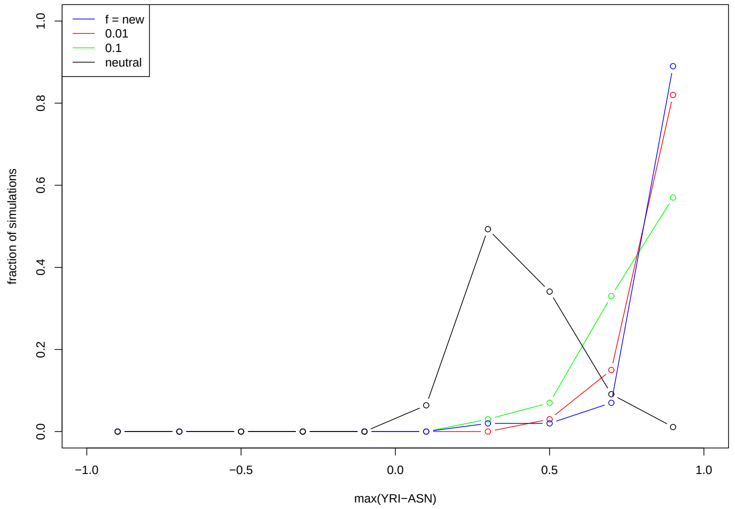


Supplementary Figure 16: **Distribution of allele frequency differences under a model of selection from standing variation**. Using the *cosi* model of human demography, we simulated populations experiencing selection only in the African branch. In each simulation, we simulated a 100kb region evolving neutrally until the African/non-African population split. At that point, we either chose a previously neutral allele at some frequency to be under selection, or we added a new selected site. We simulated three selective scenarios: one where the selected allele was present at 10% frequency prior to the change in selective pressure, one where it was present at 1% frequency, and one where we added a new selected allele. The selection coefficient was 1% in all simulations, and we simulated 100 instances of each scenario. At the end of each simulation, SNPs were thinned to the HapMap SNP density as described in the main text. For comparison, we simulated 100kb of neutral sequence using the same demography.

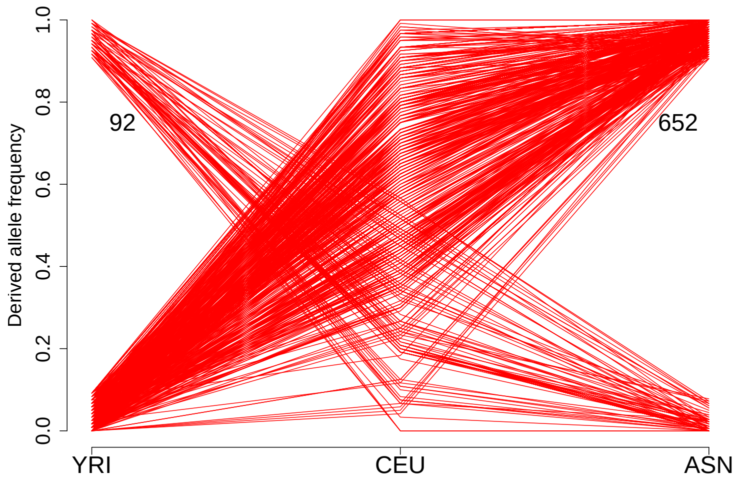


Supplementary Figure 17: **Derived allele frequencies of all SNPs with extreme frequency differences ($> 90\%$) between the YRI and ASN HapMap populations.** Each red line indicates the derived allele frequencies of a single SNP in the HapMap YRI, CEU, and ASN population groups (x-axis, left to right). The data consist of all HapMap SNPs genotyped in all three populations (irrespective of the number of centers they were typed in). The left and right numbers indicate the number of SNPs showing high derived frequency in the YRI and ASN population respectively.

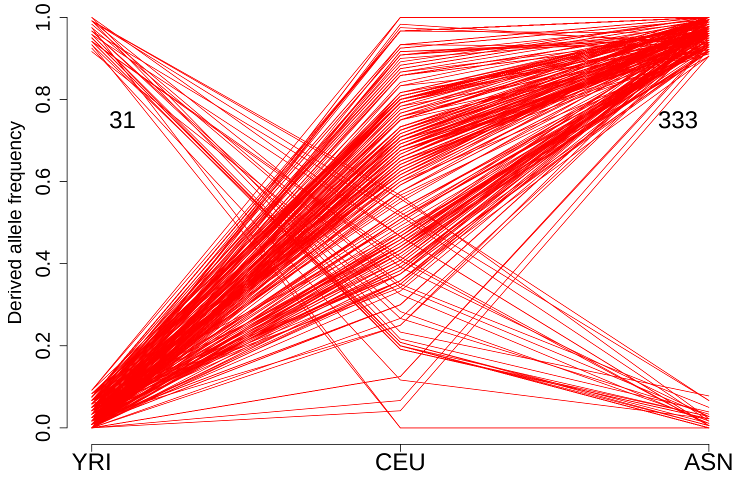


Supplementary Figure 18: **Derived allele frequencies of SNPs with extreme frequency differences (> 90%) between the YRI and ASN HapMap populations.** Each red line indicates the derived allele frequencies of a single SNP in the HapMap YRI, CEU, and ASN population groups (x-axis, left to right). The data consist of all HapMap SNPs genotyped in all three populations by two or more centers. The left and right numbers indicate the number of SNPs showing high derived frequency in the YRI and ASN population respectively.

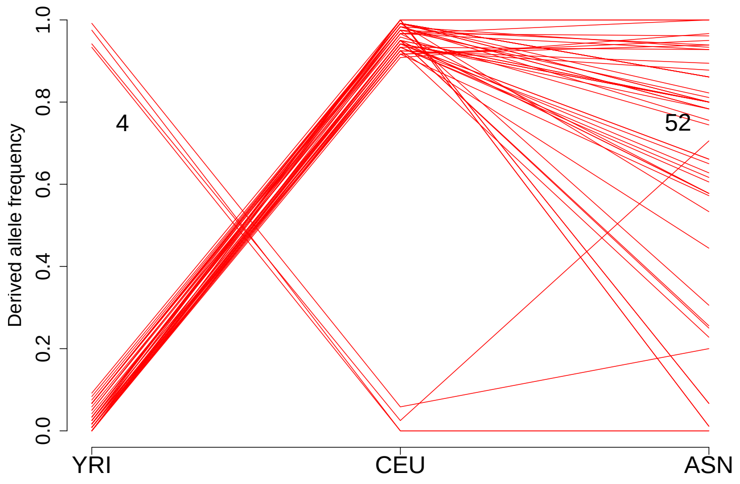


Supplementary Figure 19: **Derived allele frequencies of SNPs with extreme frequency differences ($>90\%$) between the YRI and CEU HapMap populations.** Each red line indicates the derived allele frequencies of a single SNP in the HapMap YRI, CEU, and ASN population groups (x-axis, left to right). The data are all HapMap SNPs genotyped in all three populations by two or more centers. The left and right numbers indicate the number of SNPs showing high derived frequency in the YRI and CEU population respectively.

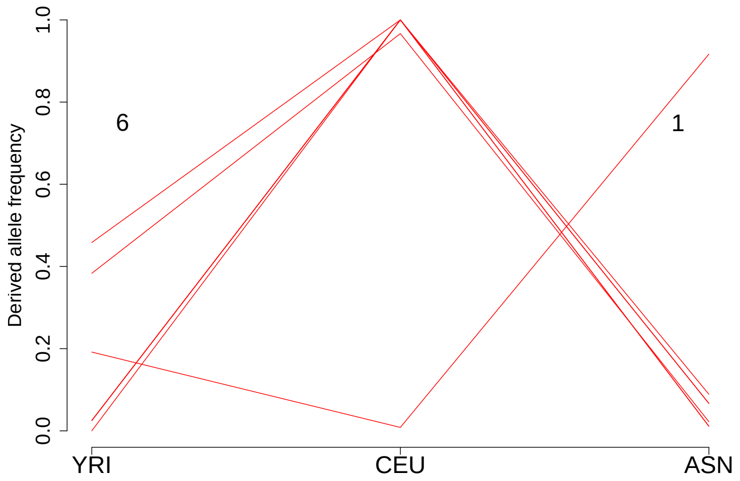


Supplementary Figure 20: **Derived allele frequencies of SNPs with extreme frequency differences ($>90\%$) between the CEU and ASN HapMap populations.** Each red line indicates the derived allele frequencies of a single SNP in the HapMap YRI, CEU, and ASN population groups (x-axis, left to right). The data are all HapMap SNPs genotyped in all three populations by two or more centers. The left and right numbers indicate the number of SNPs showing high derived frequency in the CEU and ASN population respectively.

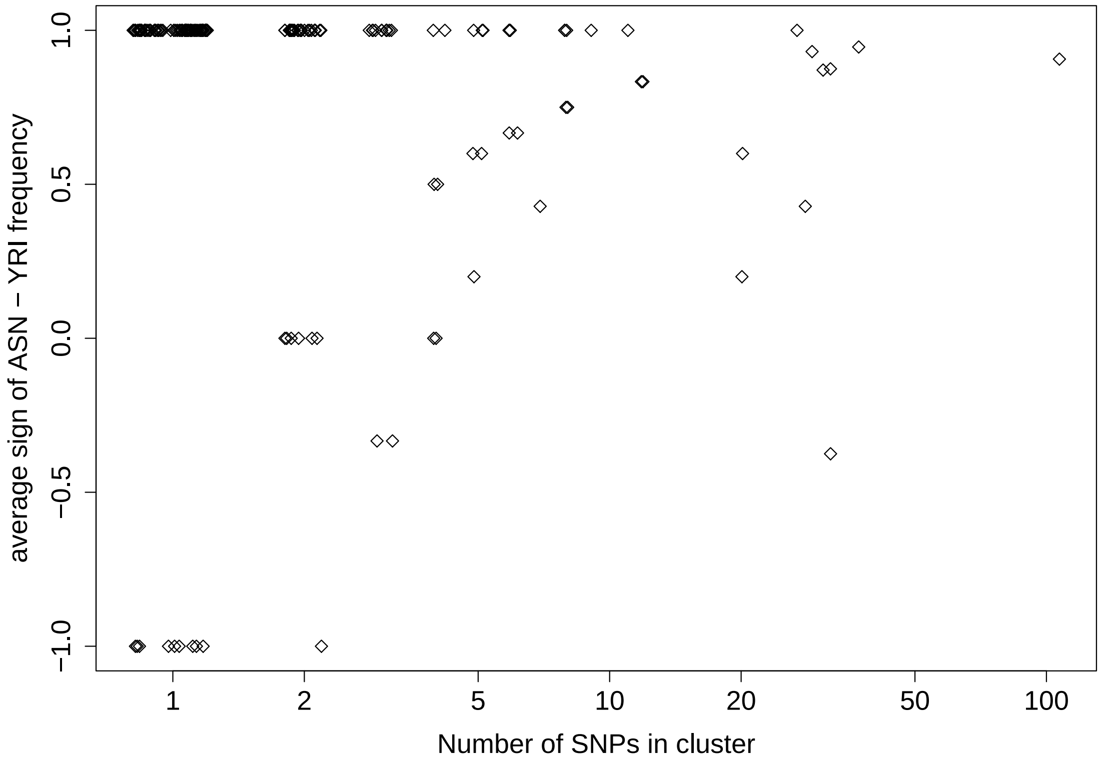


Supplementary Figure 21: **The size and population origin of clusters of highly differentiated HapMap Phase II SNPs.** Two statistics describing the 175 clusters of highly differentiated HapMap Phase II SNPs with > 90% frequency difference between YRI and ASN (see Supplementary Section 'Ancestral alleles hitchhiking with the selected variants'). We plot the number of highly differentiated SNPs in each cluster (on a log axis, with slight jitter) against the average sign of the ASN-YRI difference in derived allele frequency of SNPs in the cluster. Clusters plotted at greater than zero on the y axis are clusters where the majority of highly differentiated SNPs have a high frequency derived alleles in ASN, while in clusters below zero the majority of highly differentiated SNPs have a high frequency derived alleles in YRI. As can be seen there are few clusters of highly differentiated SNPs where the majority of SNPs in the cluster have a high derived allele frequency in YRI.

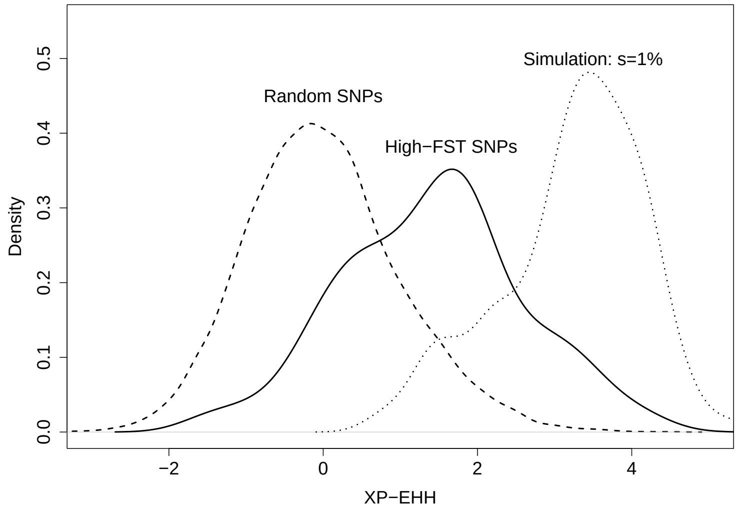


Supplementary Figure 22: **XP-EHH distribution for SNPs with $> 90\%$ frequency difference between ASN and YRI, including only SNPs that were genotyped by more than one center in the HapMap.** We cluster these extreme SNPs and use only the most extreme SNP per cluster as described in the main text.

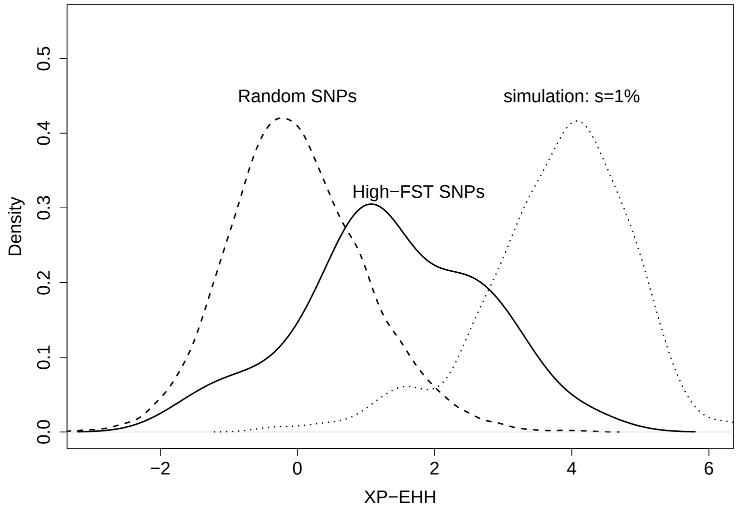


Supplementary Figure 23: **XP-EHH distribution for SNPs with $> 90\%$ frequency difference between CEU and YRI, including only SNPs that were genotyped by more than one center in the HapMap.** We cluster these extreme SNPs and use only the most extreme SNP per cluster as described in the main text.

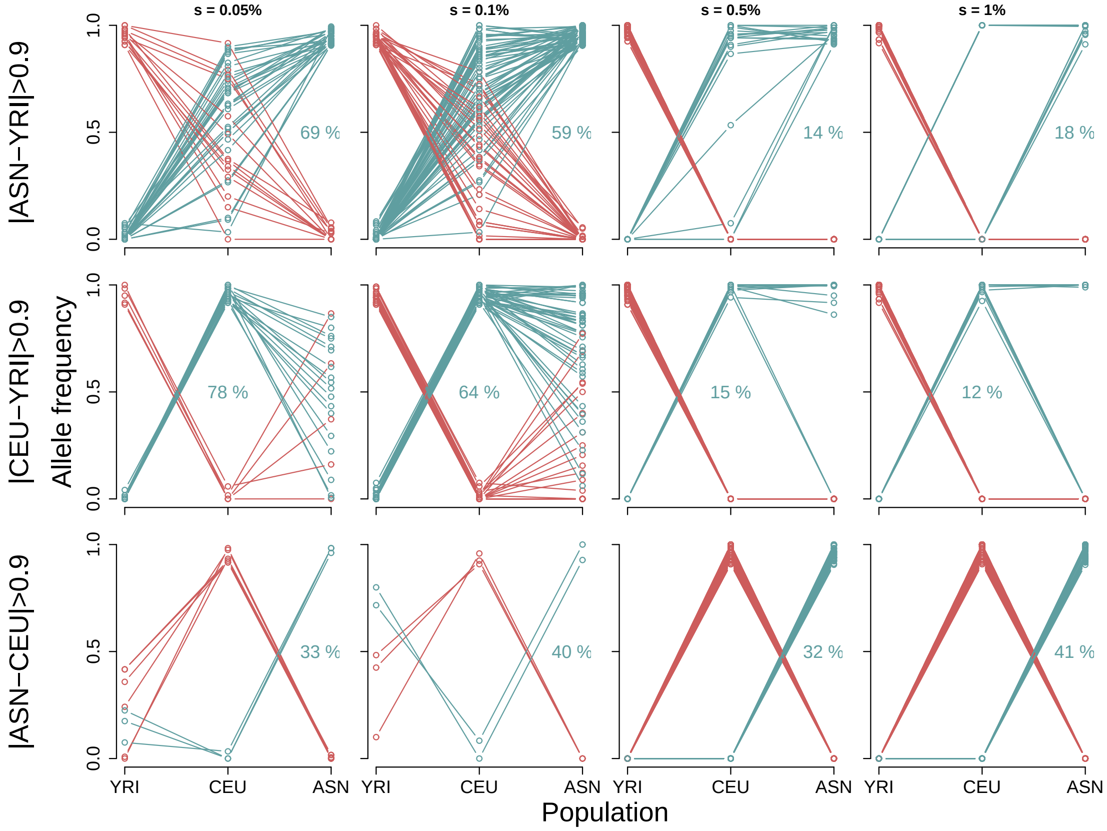


Supplementary Figure 24: **The derived frequency seen at SNPs with $> 90\%$ frequency difference in simulations with different selection coefficients**. Simulations were performed using the modified *cosi* model as described in the main text. Lines which have a high derived allele frequency in the first population in the pair are shown in blue, and in the second population in red. The number indicates the percentage of SNPs on the graph that have a high derived allele frequency in the first population in the pair.

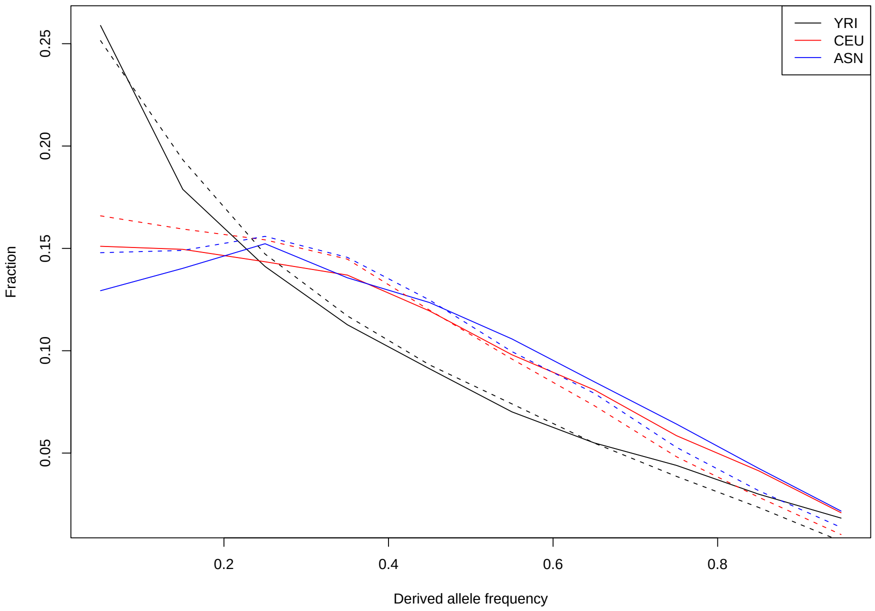


Supplementary Figure 25: **Comparison of the simulated allele frequency spectrum and Keinan et al. [9].** The frequency spectrum in the data presented in Keinan et al. [9] for the three HapMap populations (solid lines) and data generated in our implementation of the *cosi* model, and ascertained in the same manner (dashed lines). See Supplementary Section 'Assessment of the *cosi* model of human demography' for more detail.

# References


[1] Schaffner S, Foo C, Gabriel S, Reich D, Daly M, et al. (2005) Calibrating a coalescent simulation of human genome sequence variation. Genome Res 15:1576–1583.

[2] Balding D (2003) Likelihood-based inference for genetic correlation coefficients. Theor Popul Biol 63:221–230.

[3] Beaumont M (2005) Adaptation and speciation: what can F(st) tell us? Trends Ecol Evol (Amst) 20:435–440.

[4] Scheet P, Stephens M (2006) A fast and flexible statistical model for large-scale population genotype data: applications to inferring missing genotypes and haplotypic phase. Am J Hum Genet 78:629–644.

[5] Conrad D, Jakobsson M, Coop G, Wen X, Wall J, et al. (2006) A worldwide survey of haplotype variation and linkage disequilibrium in the human genome. Nat Genet 38:1251–1260.

[6] Voight B, Kudaravalli S, Wen X, Pritchard J (2006) A map of recent positive selection in the human genome. PLoS Biol 4:e72.

[7] Boyko A, Williamson S, Indap A, Degenhardt J, Hernandez R, et al. (2008) Assessing the evolutionary impact of amino acid mutations in the human genome. PLoS Genet 4:e1000083.

[8] Hawks J, Wang E, Cochran G, Harpending H, Moyzis R (2007) Recent acceleration of human adaptive evolution. Proc Natl Acad Sci USA 104:20753–20758.

[9] Keinan A, Mullikin J, Patterson N, Reich D (2007) Measurement of the human allele frequency spectrum demonstrates greater genetic drift in East Asians than in Europeans. Nat Genet 39:1251–1255.
